# Regional differences in thermal adaptation of a cold-water fish *Rhynchocypris oxycephalus* revealed by thermal tolerance and transcriptomic responses

**DOI:** 10.1038/s41598-018-30074-9

**Published:** 2018-08-03

**Authors:** Dan Yu, Zhi Zhang, Zhongyuan Shen, Chen Zhang, Huanzhang Liu

**Affiliations:** The Key Laboratory of Aquatic Biodiversity and Conservation of Chinese Academy of Sciences, Institute of Hydrobiology, Chinese Academy of Sciences, Wuhan, 430072 P.R. China

## Abstract

Understanding how populations adapt to different thermal environments is an important issue for biodiversity conservation in the context of recent global warming. To test the hypothesis that populations from southern region are more sensitive to climate change than northern region in cold-water species, we determined the thermal tolerance of two geographical populations of a cold-water fish, *Rhynchocypris oxycephalus*: the Hangzhou population from southern region and the Gaizhou population from northern region, then compared their transcriptomic responses between a control and a high temperature treatment. The results showed that the thermal tolerance range and thermal tolerance polygon area of Hangzhou population were narrower than the Gaizhou population, indicating populations from southern region were possibly more vulnerable. Further transcriptomic analysis revealed that the Gaizhou population expressed more temperature responding genes than the Hangzhou population (583 VS. 484), corresponding with their higher thermal tolerance, while some of these genes (e.g. heat shock protein) showed higher expression in the Hangzhou population under control condition, suggesting individuals from southern region possibly have already responded to the present higher environmental temperature pressure. Therefore, these results confirm the prediction that populations from southern region are more sensitive to global warming, and will be important for their future conservation.

## Introduction

Thermal adaptation is one of the most important factors that influence the fates of species in response to recent global warming^1,2^. Comparisons across plants and animals have shown some species and populations are more heat tolerant than others^3,4^, and latitudinal gradients in temperatures may result in adaptive divergence among populations^5–7^. Consequently, it is important to determine whether geographically distinct populations vary in thermal tolerance when assessing the potential impact of climate change^8^. When regional differences occur, understanding the underlying molecular mechanisms is important to better explain contemporary biogeographic patterning and predict how populations will respond to global warming^9–11^. Many studies have found intraspecific differences in thermal tolerance among populations in eurythermal species (poikilothermic species able to tolerate a wide range of body temperatures)^12–15^, but little is known to cold-water species^16,17^.

There were two mechanisms underlying thermal adaptation: phenotypic plasticity and local adaptation^9,11^. Two predictions have been made in regards to how these two mechanisms may influence species that spanning large-scale latitudinal gradients of temperature: phenotypic plasticity should correlate with the amplitude of thermal variability (the climatic variability hypothesis)^18^, or local adaptation commonly correlates with mean environmental temperature^19^. Some studies have shown support for the climatic variability hypothesis^20^. For example, higher-latitude populations of porcelain crab (*Petrolisthes violaceus*) exhibited broader thermal tolerance than lower-latitude populations^21^. Similarly, Kuo & Sanfor^22^ also found evidence in intertidal snails that populations originating from the southern range edge were the least tolerant of high temperatures^22^, whereas populations from northern regions were more warm-adapted. Those results implied that warm-adapted populations from southern regions in the northern hemisphere are most threatened by further increases in temperature because (i) their thermal limits is close to the current environmental thermal maxima, and (ii) they have only limited ability to modify thermal tolerance through plasticity^23^. In contrast, some studies have shown evidence of local adaptation that thermal tolerance is inversely correlated with latitude^24–26^. For example, in common killifish (*Fundulus heteroclitus*), the critical thermal maxima (CT_Max_) was significantly higher in southern than in northern fish by 1.5 °C at all acclimation temperatures^27^.

At the molecular level, changes in gene expression play an important role in physiological resilience to thermal stress^14,28,29^. Recent evidence in corals, gobies and barramundi suggested that increased expression of molecular chaperones such as heat shock protein (*Hsp*) genes can repair heat-induced cellular damage^25,30,31^. More studies have linked transcriptomic responses to organismal thermal tolerance to better explore the mechanisms of thermal adaptation^32^. In the marine snail (*Chlorostoma funebralis*), Gleason & Burton^26^ demonstrated that southern populations had higher thermal tolerance to heat stress than northern ones^26^, and further transcriptomic analysis revealed that southern populations may employ two strategies to cope with heat stress: upregulation of genes in response to stress and preadaptation of gene expression, but northern populations showed 23.8% more differentially expressed genes (DEGs) than the southern ones^8^. By contrast, the northern population of Australian barramundi exhibited higher tolerance to high temperatures than the southern one, and the northern population expressed 521 DEGs following heat stress while the southern one had only 171 genes^25^. Another transcriptomic study on populations of redband trout (*Oncorhynchus mykiss gairdneri*, cold-water species) revealed fish from the hot-adapted population (desert strain) had more DEGs than those from the cool-adapted population (montane strain) under thermal stress^33^. Although more and more studies began to investigate the gene expression differences between populations responding to high temperature challenges, few have targeted the cold-water species from different regions.

Chinese minnow, *Rhynchocypris oxycephalus*, is a small cold water cyprinid fish, widely distributed in East Asia. They live in cold, running, or still (but well oxygenated) waters, generally inhabiting stream headwaters at high altitude. In China, they distribute from northern Liaohe River (40.24°N) to southern Minjiang River (27.95°N)^34,35^. Across this large latitudinal range, populations experience significantly different maximum, minimum, and average air and water temperatures. Ecological modeling analysis predicted that *R*. *oxycephalus* would be sensitive to future climate change, with the most suitable habitat for the species shrinking over time especially in south-eastern China^36^. In addition, significant genetic divergence had occurred between northern and southern lineages from 2.84 Ma due to the restricted dispersal and gene flow^37^. Therefore, as a broadly distributed species with a complex phylogeographic history, these geographically distinct populations have persisted long time to cope with and adapt to the different thermal environments, which can work as an indicator species to study the intraspecific variation in thermal tolerance and the underlying molecular mechanisms in resistance to different environmental challenges.

Next-generation RNA sequencing allows for the profiling of large quantities of expression data from many samples simultaneously, where individual genes or pathways can be identified and examined in response to an experimental hypothesis^38^. This methodology is ideal for investigating the molecular mechanisms of thermal adaptation as the entire transcriptome can be examined^25^. In the current study, two geographical populations (Hangzhou population from southern region and Gaizhou population from northern region) were acclimated under four temperatures (14, 19, 24 and 29 °C) for two weeks to determine their thermal tolerance using the critical thermal methodology (CTM); then RNA-seq was performed to compare their transcriptomic responses under a control condition (19 °C) and a high temperature treatment (HTT, 29 °C). The objective is to test the hypothesis that populations from southern regions are more sensitive to climate warming than those from northern regions. Finally, we proposed suitable conservation strategies based on our results.

## Results

### Thermal tolerance of Hangzhou and Gaizhou populations

Both CT_Max_ and critical thermal minima (CT_Min_) values increased with increasing acclimation temperatures in the two populations (Table 1). At each acclimation temperature (14, 19, 24 and 29 °C), the mean CT_Max_ of the Gaizhou population was a little higher than the Hangzhou population but without significant difference (*P* > 0.05, Table 1). However, the mean CT_Min_ of the Gaizhou population was significantly lower than the Hangzhou population under the acclimation temperatures of 24 and 29 °C (*P* < 0.05, Table 1). Therefore, the thermal tolerance range of Gaizhou population (32.01–33.93 °C) was wider than the Hangzhou population (30.44–33.13 °C, Table 1), and the thermal tolerance polygon area of the Gaizhou population (498.88 °C^2^) was broader than the Hangzhou one (478.98 °C^2^, see Supplementary Fig. S1).

**Table 1:** Thermal tolerance of *R. oxycephalus* at different acclimation temperatures.

Acclimation temperature (°C)	Population	*CT*_Max_ (°C) Mean ± SD	*CT*_Min_ (°C) Mean ± SD	Thermal tolerance range (°C)
14	Hangzhou	32.29 ± 0.29^d^	0.00 ± 0.00^e^	32.29
	Gaizhou	32.40 ± 0.23^c,d^	0.00 ± 0.00^e^	32.40
19	Hangzhou	33.23 ± 0.11^c^	0.10 ± 0.00^e^	33.13
	Gaizhou	33.93 ± 0.06^c^	0.00 ± 0.00^e^	33.93
24	Hangzhou	33.40 ± 0.09^c^	2.10 ± 0.00^d^	31.30
	Gaizhou	33.99 ± 0.10^c^	1.31 ± 0.12^c^	32.68
29	Hangzhou	35.71 ± 0.09^a,b^	5.27 ± 0.10^b^	30.44
	Gaizhou	35.75 ± 0.12^a^	3.76 ± 0.12^a^	32.01

Values with different superscript letters (a,b,c,d,e) within a column indicates significant difference (ANOVA, P < 0.05).

### De novo assembly and functional annotation

Due to the absence of a reference genome for *R*. *oxycephalus*, a transcriptome was *de novo* assembled as a reference for gene expression profiling. There was 84.84 Gb clean reads with an average of 7.07 Gb clean reads for each sample. Finally, 155,636 unigenes were identified as orthologs.

### Transcriptomic response under control condition and HTT

**Figure 1:**
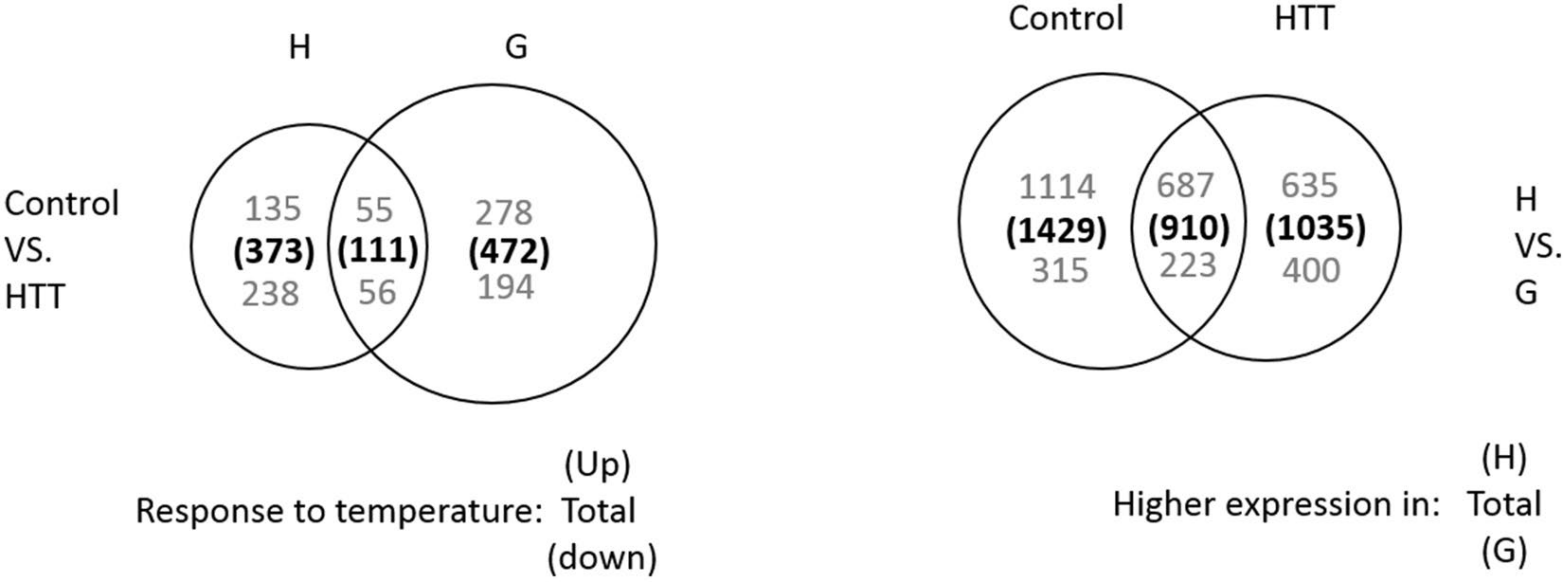
Venn diagram showing the number of differentially expressed genes identified during analysis based on within-population temperature response (HTT, high temperature treatment), and within-treatment population differences. Bold numbers indicate totals and respective shades of grey indicate upregulated vs. downregulated or higher expression in Hangzhou (H) vs. Gaizhou (G) regions, respectively.

Under control condition, individuals from the Hangzhou and Gaizhou populations differed significantly in gene expression across 2339 genes (1801 higher in the Hangzhou population, and 538 higher in the Gaizhou population) (Fig. 1). Categorical classification of these 1801 genes were found to be enriched in regulation of binding (retinoid, carbohydrate binding, etc.), metabolic process and catalytic activity, while 538 were enriched in binding (metal ion binding, etc.) and cellular process. Under HTT, the Hangzhou and Gaizhou populations differed at 1945 genes, 1322 were greater in the Hangzhou population and 623 were higher in the Gaizhou ones (Fig. 1). This phenomena showed that the number of higher expressed genes in the Hangzhou population was decreased (enriched in binding) after HTT while increased (enriched in immune system process) in the Gaizhou population. Among the DEGs under control condition and after HTT, 910 genes were identical, and 687 were always expressed higher in the Hangzhou population and 223 were always expressed greater in the Gaizhou one (Fig. 1).

To validate the DEGs obtained from RNA-seq, we selected eight genes for qRT-PCR analysis. As shown in Supplementary Fig. S2, the qRT-PCR expression patterns of five genes matched the results detected by RNA-seq in the Hangzhou population, and the other three genes (*GGCTB*, *Dnaj1*, *Hsp90*) did not have a perfect match. Similarly, seven genes matched the results detected by RNA-seq in the Gaizhou population except the *GGCTB*. Overall, the qRT-PCR results were consistent with the RNA sequencing data, indicating that our transcriptome data were credible.

### Common transcriptomic responses between Hangzhou and Gaizhou populations after HTT

After two weeks of HTT, a large number of temperature responding genes (TRGs) were found in the comparisons of HTT and control condition in both populations, as visible in the PCA (see Supplementary Fig. S3). There were 111 genes exhibiting identical expression change patterns after HTT in Hangzhou and Gaizhou populations: 55 were upregulated and 56 downregulated (Fig. 1). However, although these 111 genes were expressed in both populations, the magnitude of upregulation and downregulation was often different between the two populations. The most highly upregulated of these 111 genes found in both populations in the comparison of HTT and control condition was heat shock protein 70 (*Hsp*70), which can play a significant role in heat stress. Another two members of the *Hsp* family (*Hsp*90a, *Hsp*90a.2) were also significantly upregulated in both populations. The most significantly downregulated gene was not annotated, but the second was a zinc finger protein *Aebp2*, with putative roles in DNA-binding transcriptional repressor.

### Specific transcriptomic responses to HTT in Hangzhou and Gaizhou populations

**Figure 2:**
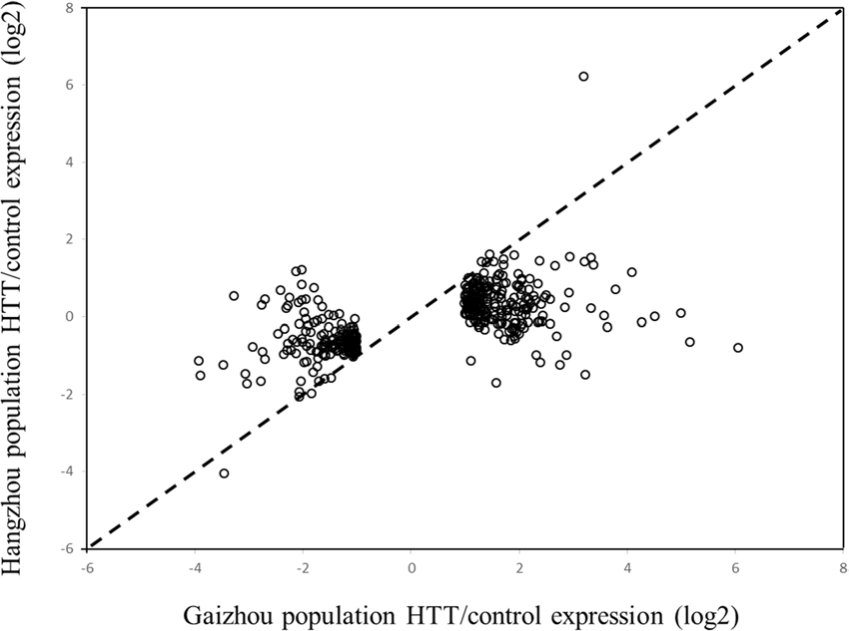
Scatterplot of the log2 fold changes in gene expression in response to high temperature treatment (HTT) in the Gaizhou population vs. the Hangzhou population for the 472 differentially expressed genes that were unique to the Gaizhou population. Each open circle represents an individual uinigene, the dashed line is a 1:1 line.

**Figure 3:**
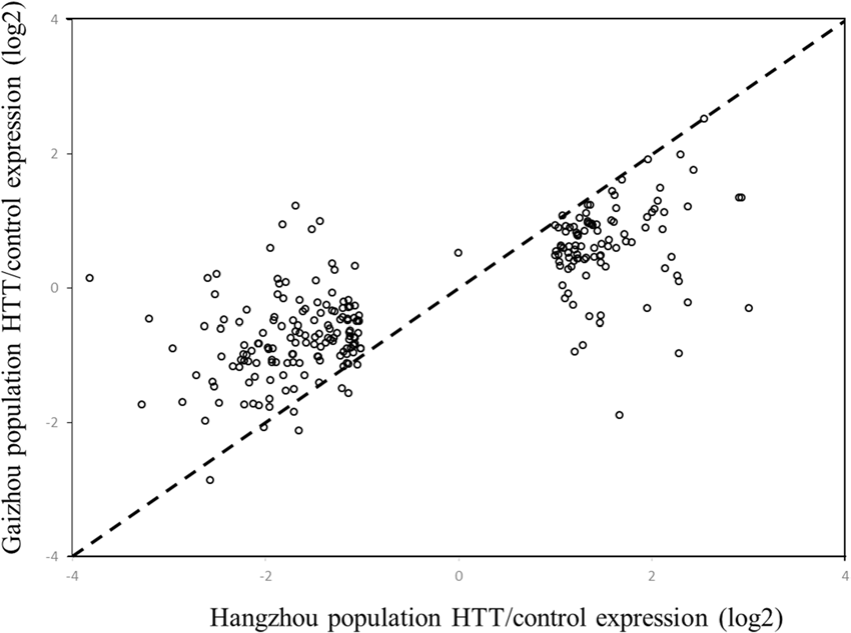
Scatterplot of the log2 fold changes in gene expression in response to high temperature treatment (HTT) in the Hangzhou population vs. the Gaizhou population for the 373 differentially expressed genes that were unique to the Hangzhou population. Each open circle represents an individual unigene, the dashed line is a 1:1 line.

Different transcriptomic responses reacting to HTT between the two populations were found in several ways. First, excepting the 111 identically responded genes, each population had hundreds of genes which reached significantly different level after HTT in one population but did not significantly change in another one. However, most of them exhibited the same upregulated or down regulated pattern. For example, 472 genes reacted significantly to HTT in the Gaizhou population but did not significantly change in the Hangzhou population (Fig. 1). Further inspection of the data showed that 371 of these 472 (79%) genes responded in a similar pattern with a lesser magnitude of regulation in the Hangzhou population (Fig. 2), although significance was precluded by more variable genes. Among these 472 TRGs, 278 were significantly upregulated and 194 were downregulated after HTT in the Gaizhou population. These 278 upregulated genes did not significantly changed in the Hangzhou population after HTT, however, 195 of 278 genes (70.1%) had higher expression under control condition when compared with the Gaizhou one (Supplementary Table S2). Similarly, 194 downregulated genes did not significantly changed in the Hangzhou population after HTT, while 162 of 194 genes (83.5%) showed lower expression under control condition when compared with the Gaizhou one (Supplementary Table S3). In other words, many genes that significantly upregulated in the Gaizhou population after HTT have already showed higher expression under control condition in the Hangzhou population, and that many significantly downregulated genes in the Gaizhou population have already responded lower expression under control condition in the Hangzhou one. By contrast, 373 genes were found significantly responding to HTT in the Hangzhou population but not in the Gaizhou one (Supplementary Tables S4 and S5). Most of these genes (232 in 373, 62%) did not reach the significant level in the Gaizhou population, and lack of significant change is a result of lesser magnitude in expression (Fig. 3).

Second, the Hangzhou population showed 20% less TRGs than the Gaizhou population (484 vs. 583; Fig. 1). For example, the Hangzhou population had only three ubiquitin protein genes that expressed differentially after HTT while the Gaizhou population had eight this kind of genes. Two hemoglobin genes (hemoglobin beta chain, hemoglobin subunit alpha-1) were significantly downregulated in the Gaizhou population after HTT but was not significantly changed in the Hangzhou population. Categorical classification of these genes showed that the 583 Gaizhou population TRGs were enriched in the function of binding, cellular process and single-organism process, while the 484 Hangzhou population TRGs were enriched in binding, cellular process and metabolic process.

### Population-specific responses to high temperature: Hsps

After HTT, a class of *Hsp* genes exhibited prominent upregulation including six major families (s*Hsp*, *Hsp*40s, *Hsp*47, *Hsp*60, *Hsp*70s and *Hsp*90s) in both the two populations (Table 2). However, the pattern of these significant upregulations (i.e. more upregulated in the Gaizhou or Hangzhou population) was unique to the particular *Hsp* gene family. For instance, four of the five (80%) annotated *Hsp*90s, *Hsp*60 and s*Hsp* showed a higher fold change after HTT in the Gaizhou population compared with the Hangzhou population. In contrast, most *Hsp*70s (4 in 6, 67%) and all dnaj (*Hsp*40) genes were more highly expressed in the Hangzhou population following HTT.

**Table 2:** The average expression values of major heat shock protein-related genes.Hsps.

	Unigene_ID	Nr_annotation	H19	H29	G19	G29	*P-value*
Hsp90s	c70782.graph_c0	Heat shock protein 90-alpha-like	216	1736	132	1074	6.90E-02
	c92050.graph_c0	Heat shock protein 90 kda beta member 1	2124	4589	1849	3993	0.92
	c73802.graph_c0	Heat shock protein 90a.2 protein	498	4176	288	3448	0.77
	c71586.graph_c0	Heat shock protein HSP 90-alpha 1	331	3100	231	2254	0.29
	c84983.graph_c0	Activator of 90 kda heat shock protein	613	1708	717	1540	0.35
Hsp70s	c78321.graph_c0	Heat shock protein 4a	589	1514	601	1553	0.15
	c92413.graph_c0	Heat shock protein 9	3151	6385	2944	5592	0.51
	c86125.graph_c0	Heat shock protein 105 kda isoform X3	564	1915	280	1154	2.49E-05
	c90157.graph_c0	Heat shock protein 70#1	248	6332	1021	4541	0.39
	c72419.graph_c0	Heat shock protein 70#2	92	228	56	122	2.05E-03
	c53548.graph_c0	Heat shock protein 70#3	97	288	79	160	2.25E-03
Hsp40s	c72381.graph_c0	Dnaj homolog subfamily A member 1#1	340	751	335	704	0.18
	c80217.graph_c0	Dnaj homolog subfamily A member 1#2	55	884	128	861	0.27
	c92400.graph_c0	Dnaj homolog subfamily B member 1	149	640	287	532	0.77
	c89579.graph_c0	Dnaj homolog subfamily B member 1-like	299	817	405	860	1.26E-02
Hsp60	c91023.graph_c0	60 kda heat shock protein	1967	4245	2098	4772	2.54E-05
Hsp47	c84339.graph_c0	Heat shock protein 47	230	990	229	551	4.72E-02
sHsp	c95195.graph_c0	Alpha-crystallin B chain-like	753	2446	306	1149	2.25E-05
Others	c88517.graph_c0	Heat shock transcription factor 2c	1152	2649	962	2435	0.69
	c93785.graph_c1	Heat shock factor protein 1	576	1977	450	1068	3.10E-03
	c79323.graph_c0	Stress-induced-phosphoprotein 1	1256	2804	1273	2618	0.20

H19 meaning the Hangzhou population at 19 °C; H29 meaning the Hangzhou population at 29 °C; G19 meaning the Gaizhou population at 19 °C; G29 meaning the Gaizhou population at 29 °C.

### KEGG pathways enriched in high temperature stress regulated genes

**Figure 4:**
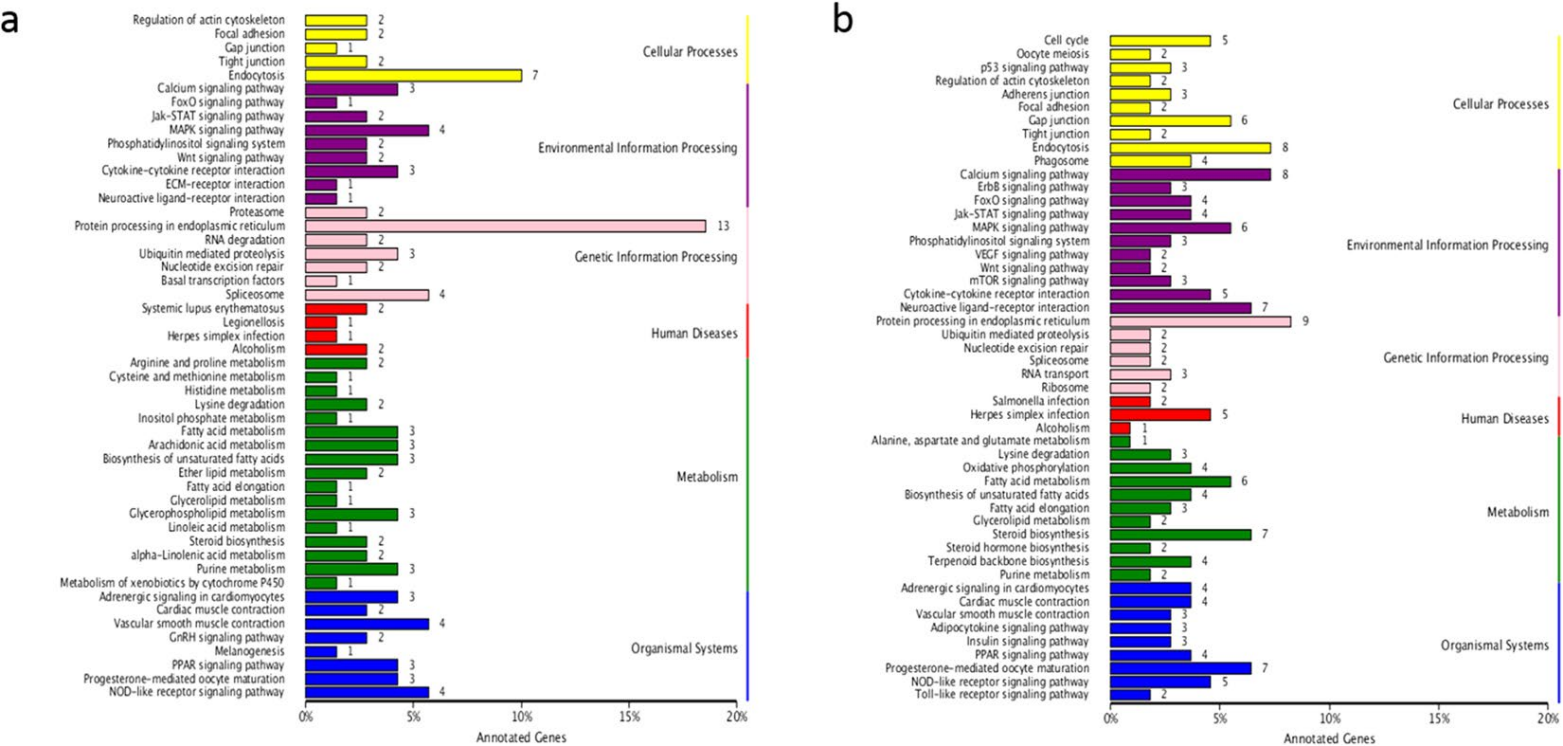
KEGG classification after high temperature treatment (HTT) in (**a**) Hangzhou population; (**b**) Gaizhou population.

The KEGG pathway analysis revealed protein processing in endoplasmic reticulum and endocytosis as the two most highly enriched pathways after HTT in both the two populations. Excepting these two pathways, the highest number of TRGs (≥4) was enriched in 20 other pathways in the Gaizhou population such as calcium signaling pathway, neuroactive ligand-receptor interaction and steroid biosynthesis (Fig. 4). In the Hangzhou population, the highest number of TRGs (≥4) was enriched in only three pathways including NOD-like receptor signaling pathway, spliceosome, progesterone-mediated oocyte maturation and MAPK signaling pathway (Fig. 4). Therefore, the Gaizhou population had more TRGs enriched in signal transduction pathways than the Hangzhou population.

## Discussion

In the current study, mechanisms of temperature adaptations were examined by comparing thermal tolerance and transcriptomic responses to high temperature between two geographical populations in a cold-water species *R*. *oxycephalus*. The results demonstrated that the southern population (Hangzhou) has narrower thermal tolerance range than the northern population (Gaizhou), suggesting populations from southern regions are more heat sensitive to high temperature. Their divergent transcriptomic responses also implied their regional differences in gene expression reacting to HTT: the northern population (Gaizhou) may have more potential genes responding to high temperature, which results in their broader thermal tolerance range, while the southern population (Hangzhou) have many genes already responded to the higher environmental temperature which results in their narrower thermal tolerance range. Of course, these results will be much stronger if more replicate populations from northern and southern regions are included rather than a single population for each site.

The thermal tolerance experiments indicated that the southern population (Hangzhou) exhibited a little lower CT_Max_ and possessed narrower thermal tolerance range than the northern one (Gaizhou), suggesting a trend that the southern population (Hangzhou) was more heat sensitive than the northern one (Gaizhou). In other words, the northern population (Gaizhou) had a little higher CT_Max_ and broader thermal tolerance range. This result is consistent with the climatic variability hypothesis that individuals at higher latitudes (northern regions) require broader tolerance ranges (i.e. greater physiological plasticity) than individuals inhabiting lower latitudes (southern regions)^22^, because theory suggests that the thermal adaptation of many organisms are proportional to the magnitude of variation in environmental temperature they experience^39,40^. This positive correlation of thermal tolerance and latitude has also been described in other ectotherms, predicting that at low latitudes (southern regions) rising environmental temperature, which is similar to global warming, is likely to have the most deleterious consequences because ectothermic species in these geographic areas are in greatest danger from further increases in environmental temperature^20,41,42^. Furthermore, because low latitudinal (southern) populations may have higher metabolic rates, thermal acceleration of metabolism by rising temperatures has a higher absolute effect on rates of metabolic turnover than in high latitudinal (northern) populations with intrinsically lower rates of metabolism^43^. Therefore, our results confirmed the prediction that the southern population (Hangzhou) of *R*. *oxycephalus* are becoming increasingly vulnerable as global warming threaten their future sustainability, therefore conservation efforts should pay more attention to those populations to avoid local extinction.

Higher expression under control condition and lesser regulation after HTT for many genes in the southern population (Hangzhou) may represent a degree of already response to resist the higher environmental temperature they have experienced. In this study, 195 of 278 genes which were significantly upregulated in the northern population (Gaizhou) after HTT, had higher expression under control condition when compared with the northern one (Gaizhou), while 162 of 194 genes which were significantly downregulated in the northern population (Gaizhou) after HTT, showed lower expression under control condition when compared with the northern one (Gaizhou) (Supplementary Tables S2 and S3). We propose that these TRGs (195 or 162) have already responded to the higher environmental temperature (higher or lower expression under control condition) in the southern population (Hangzhou), and they did not significantly regulate in the southern population (Hangzhou) after HTT. Normally, the southern population (Hangzhou) of *R*. *oxycephalus* have been inhabited in lower-latitude regions for a long period (about 2.84 Ma)^37^, where they experienced higher mean water temperature than the northern one (Gaizhou), so they have already expressed some responded genes to resist their higher living temperature, which is consistent with the result that 687 genes were always expressed higher level in the southern population (Hangzhou). This result seems contrary to the results studied in the snail and coral. In marine snail (*C*. *funebralis*), the southern population have higher thermal tolerance to heat stress than northern ones, but southern populations exhibited the similar pattern in this study that some genes showed higher expression under control condition implying preadaptation of gene expression^8^. The heat-tolerant coral populations also had constitutive frontloaded genes to maintain their physiological resilience^31^. We suggest that these already responded genes in the southern population (Hangzhou) of *R*. *oxycephalus* may imply they are already under the thermal stress possibly because of it’s a cold-water species, however, preadaptation of genes in snails or corals is a result of local thermal adaptation.

Differential gene expression may result in regional differences in thermal tolerance. Comparative transcriptomic analysis identified 1801 genes expressed higher level in the southern population (Hangzhou) and 538 were higher in the northern one (Gaizhou) under control condition, while these genes became 1322 and 623 after HTT, respectively. Among these differentially expressed genes under control condition and after HTT, 687 were always expressed higher in the southern population (Hangzhou) and 223 were always expressed greater in the northern population (Gaizhou) (Fig. 1). Besides, the northern population (Gaizhou) had 20% more TRGs (583 vs. 484) than the southern population (Hangzhou) after HTT. These data may reflect that the northern population (Gaizhou) have more potential genes to regulate for response than the southern one (Hangzhou) when given the same thermal event (Fig. 1). This result imply an important molecular mechanism that the larger number of TRGs may lead to their higher thermal adaptation. Similar pattern has been detected in many previous transcriptomic studies^8,25,43^. For example, the redband trout (*O*. *mykiss*, cold-water species) from the desert population (heat tolerant) had more expressed transcripts than those from the montane population (heat sensitive) under thermal stress, suggesting a lot of genes has evolved to adapt in their desert environment^33^. Similarly, the larger number of differentially expressed genes detected in *Anolis homolechis* and *A*. *sagrei* suggested these two species were better at transcriptional regulation under the higher temperature than another sympatric species *A*. *allogus* (with lower body temperature). Furthermore, as heat shock response, more ubiquitin genes were significantly upregulated and two haemoglobin genes were significantly downregulated in the northern population (Gaizhou) after HTT, which contributing to their higher thermal tolerance range. Quinn *et al*.^44^ also found increased expression of ubiquitin in Arctic charr (*Salvelinus alpinus*) exposed to temperature stress and reported a down regulation of haemoglobin genes in fish that showed tolerance to increased temperatures^44^. By contrast, *Trematomus bernacchii*, a cold-water fish species, has been shown to be unable to mount a heat shock response despite retaining the heat shock gene *Hsp*70 and the regulation factor *Hsf*1^30^. The inability to mount or responded less magnitude in heat shock response highlights the susceptibility of these species or populations to global warming. Therefore, these data provided more evidence to prove that the southern population (Hangzhou) of *R*. *oxycephalus* may be more vulnerable to high temperature.

After HTT, all heat shock protein family genes were upregulated (Table 2), because they can help the body adapt to adverse environments by serving as molecular chaperones^45–47^. These results are consistent with previous research in other organisms that *hsp* genes are commonly identified overexpressed under high temperatures^48,49^. Although all *hsps* were upregulated, different expression patterns could be detected between the southern and northern populations. All but one of the annotated *Hsp90*s, *Hsp60* and s*Hsp* were more highly upregulated in the northern population (Gaizhou) compared with the southern individuals (Hangzhou), which consistent with the northern population’s (Gaizhou) higher thermal tolerance. While these genes might have already responded to the higher environmental temperature in the southern population (Hangzhou) (with higher expression under control condition), they did not upregulated so much when following the same HTT due to their narrower thermal tolerance range. There is some support from *Drosophila* and *Arabidopsis* that enhanced levels of *Hsps* can be deleterious^49–51^, and thus lesser magnitude of upregulation in the southern population could be protective, suggesting *Hsps* responses reacting to HTT (29 °C) in the southern population are likely a result of being under stressful conditions rather than an adaptive response to thermal stimuli. In contrast, four in six *Hsp70*s and all *Hsp40*s were more highly upregulated in the southern population (Hangzhou) following HTT compared with the northern population (Gaizhou) (Table 2). We hypothesize that these *Hsp70*s and *Hsp40*s are actually a result of the northern population’s (Gaizhou) higher thermal tolerance because they do not incur as much damage following the same HTT while the southern population (Hangzhou) upregulate greater expression of these two families genes to respond HTT. Because *Hsp*40s enhance the overall chaperone efficiency including *Hsp*70, it has been proposed that *Hsp*70 and *Hsp*40 often worked together to increase the protein thermostability and accelerated recovery from protein damage in thermotolerant cells^52–54^. This result supported this view that higher *Hsp*40s expression combined with higher *Hsp*70 expression^55^. Similar *hsps* expression pattern has also been observed in snails that southern populations (heat tolerant) showed greater upregulation for approximately half of the *Hsp40*s^8^. However, this pattern contrasts with the results that observed in the corals and tide pool copepod *Tigriopus californicus*: Barshis *et al*.^31^ found that less heat tolerant populations showed a higher expression in several s*Hsps* compared with more heat tolerant corals following heat stress^31^; Schoville *et al*.^56^ found a more thermally tolerant population upregulated several *Hsp70* paralogs to a greater extent than a less tolerant population^56^.

In this study, pathways such as “protein processing in endoplasmic reticulum,” “endocytosis”, “spliceosome,” “calcium signaling pathway,” “MAPK signaling pathway,” and “steroid biosynthesis” were enriched under thermal stress, which is consistent with those studies found in zebrafish and carp^57–59^. In the study of Yong Long *et al*.,^58^ the “spliceosome” was the most significantly enriched pathway in zebrafish under cold acclimation. We also found that “protein processing in endoplasmic reticulum spliceosome” and “spliceosome” were the two most significantly enriched pathway after HTT in both two populations. Besides, more pathways were enriched in the northern population (Gaizhou), including calcium signaling pathway, neuroactive ligand-receptor interaction and some metabolism pathways (steroid biosynthesis, fatty acid metabolism, etc.) (Fig. 4). However, the signal transduction mechanisms used by fish cells to sense heat signals and trigger intracellular responses remain largely unknown^58^. Therefore, the northern population (Gaizhou) had more TRGs enriched in more pathways such as steroid biosynthesis which is related to metabolism, while the southern one (Hangzhou) mainly enriched in the key signal transduction pathways due to their narrower thermal tolerance range.

## Methods

### Ethics statement

All experimental protocols about dealing with the fish in this study were approved by the Ethics Committee for Animal Experiments of the Institute of Hydrobiology, Chinese Academy of Sciences. The methods used in this study were conducted in accordance with the Laboratory Animal Management Principles of China.

### Sample collection and thermal tolerance experiment

Individuals of *R*. *oxycephalus* were collected in the May 2015 from northern region: Gaizhou county (40.24°N, 122.45°E, water temperature: 11.8 °C) and southern region: Hangzhou city (30.23°N, 120.09°E, water temperature: 15.6 °C) with hand nets. The temperature information of each sampling site could be obtained from the WorldClim database (available at http://www.worldclim.org) during the 1950–2000 periods. The mean diurnal range: mean of monthly (max temp–min temp) of Gaizhou (9.2 °C) is higher than Hangzhou (8.0 °C), and the temperature annual range for Gaizhou and Hangzhou is −15.1–26.8 °C and 0.8–32.9 °C, respectively. All the experimental fishes were one year old. Captured fishes were transported to the Institute of Hydrobiology, Chinese Academy of Sciences (Wuhan), and housed in the laboratory conditions (19 ± 0.2 °C) for three weeks. This temperature is close to the field temperature to ensure that fishes’ thermal history was not disturbed after collection. Fishes were kept under a natural light regime and fed with frozen commercial fish flake twice a day. Fish from each population were acclimated in thermostatic aquaria separately at four acclimation temperatures (14, 19, 24 and 29 °C). The water temperature in the aquaria was increased or decreased at a rate of 1 °C per day from the ambient temperature (19 ± 0.2 °C) to reach the treatment temperatures. After achieving the desired acclimation temperatures, fish were maintained in their respective temperatures for a period of two weeks. During the acclimation period, the water dissolved O_2_ level varied between 95% and 100%. Water exchange was carried out every day to maintain the water quality. The fish were not fed for 24 h before being subjected to the temperature tolerance.

Thermal tolerance of *R*. *oxycephalus* was evaluated by the CTM, including CT_Max_ and CT_Min_^60^. This technique is well established as a powerful tool for studying the physiology of stress and adaptation in fish^24^. The mean CT_Max_ and CT_Min_ of the Hangzhou population have been evaluated in our previous publication^61^. Therefore, the CT_Max_ and CT_Min_ of the Gaizhou population were added in this study. A total of 80 fish (10 individuals per acclimation temperature) were used for determination of thermal tolerance. Fish acclimated to a particular temperature were subjected to constant rate (1 °C/h) of increase or decrease in the water temperature until loss of equilibrium (LOE) was reached. The temperature at which LOE occurred was recorded as CT_Max_ and CT_Min_ for each fish, respectively. At the end of each trial, fish were measured (total length ± 0.1·cm), weighed (wet mass ± 0.1·g). All the fish were rescued and recovered after transfer to ambient temperature from the endpoint of CTM trial. The thermal tolerance polygon was generated by plotting the acclimation temperatures on the X-axis and the mean CT_Max_ and CT_Min_ values on the Y-axis. The thermal tolerance zone area was calculated from the polygon and expressed as °C^2^.

### RNA preparation

Individuals from the Hangzhou and Gaizhou populations were kept at the control condition (19 °C) and exposed at a high temperature treatment (HTT, 29 °C) for two weeks, respectively. Three biological replicates of both HTT and control condition were performed for each population. All samples were euthanized by soaking buffered MS-222 solution (3 g/l). Brain tissue was chosen for this experiment due to the complex physiological role and its rapid response to environmental stressors^43^. Total RNA was extracted using an RNA extraction kit (Omega Bio-Tek, USA) with Trizol® Reagent (Invitrogen, CA, USA) following the manufacturer’s instructions. Twelve high-quality RNA samples were finally acquired.

### Illumina sequencing and de novo assembly

For each sample, a total amount of 3 μg RNA was used as input material. Sequencing libraries were generated using the NEBNext® Ultra™ RNA Library Prep Kit for Illumina® (NEB, USA) following manufacturer’s recommendations. Briefly, mRNA was purified using poly-T oligo (dT) magnetic beads, and fragments were generated using divalent cations under elevated temperature in NEBNext First Strand Synthesis Reaction Buffer (5×). First strand cDNA was synthesized using random hexamer primers and M-MuLV Reverse Transcriptase (RNase H−), and second strand cDNA was subsequently synthesized with DNA Polymerase I and RNase H. Remaining overhangs were converted into blunt ends via exonuclease/polymerase activities. The library fragments with a preferential length of 150 to 200 bp were purified with the AMPure XP system (Beckman Coulter, Beverly, USA). The above cDNA samples were then clustered using the TruSeq PE Cluster Kit (Illumina, USA) on a cBot Cluster Generation System. We sequenced all libraries on an Illumina Hiseq4000 platform (San Diego, CA, USA), and 151 bp paired-end reads were then generated for each of them.

Clean reads were obtained by removing the trimming adapter sequences, reads containing poly-N and low quality reads (reads with greater than 5% unknown nucleotides or reads with less than 60 bp) from raw reads. The number of raw reads, clean reads and Q30 were calculated at the same time (Table S1). All subsequent analyses were based on the clean reads with high quality. The left files (read1 files) from all libraries/samples were pooled into one big left.fq file, and right files (read2 files) into one big right.fq file. Transcriptome assembly was accomplished based on the left.fq and right.fq using Trinity program with min_kmer_cov set to 2 and with default values for all remaining parameters^62^. Unigenes was selected from the longest transcript copy of each gene clusters to avoid redundant transcripts^63^. All unigenes from the 12 samples were combined in a unigene database for *R*. *oxycephalus*, and all subsequent analyses were performed on this database.

### Functional annotation of unigenes

To functionally annotate the unigenes in *R*. *oxycephalus*, we searched our unigene set against the datasets of nonredundant protein sequences (NR) in the National Center for Biotechnology Information (NCBI; http://www.ncbi. nlm.nih.gov), the Protein family database (PFAM; http:// pfam.xfam.org/) and the manually annotated and reviewed protein sequence database (Swiss-Prot database; http://www.ebi.ac.uk/swissprot/) using NCBI-BLAST (v 2.2.30) with an E-value cut-off of 1 × 10^−5 ^^64^. Annotation was extended to the Functional classifications of Gene Ontology (GO; http://www.geneontology.org/), the Clusters of Orthologous Groups of proteins (KOG/COG; http://www.ncbi.nlm.nih.gov/COG), and Pathway Annotation of the Kyoto Encyclopedia of Genes and Genomes (KEGG; http://www.genome.jp/kegg/pathway.html). GO annotation was performed using Blast2GO pipelines with an E-value ≤ 1E^−5^^65,66^. KOG annotation was searched with an E-value ≤ 1E-5^67^. We searched the unigenes against the KEGG database using KOBAS 2.0 with an E-value ≤ 1E^−10 ^^68^.

### Mapping and differential expression analyses

Firstly, clean reads of each brain sample were mapped back to the assembled transcriptome with the alignment program Bowtie2^69^. Bowtie2 was implemented in the −v alignment mode with the maximum number of mismatches set to 2. Paired end reads were aligned to the transcriptome with both read pairs needing a valid alignment within a given locus to be counted as a match. If more than one alignment was possible the best match was reported according to the least number of mismatches for each read and overall for the pair. Then, the read count for each gene was obtained from the mapping results, which was then used as input for the program DESeq2 R package (v2.15.3)^70^ that estimates variance-mean dependence in the data and tests for differential expression based on the negative binomial distribution. The three samples from each treatment were used to generate mean expression levels with associated variances. The false discovery rate (FDR) was controlled at 5% according to the method of Benjamini and Hochberg^71^. Genes with an adjusted *p*-value (FDR) < 0.05 and |log_2_ (fold-change)| >1 were assigned as significant level. Four pairwise comparisons were performed to examine differences in gene expression in response to HTT: (i) Hangzhou control vs. Gaizhou control, (ii) Hangzhou HTT vs. Gaizhou HTT, (iii) Hangzhou control vs. Hangzhou HTT and (iv) Gaizhou control vs. Gaizhou HTT. These respective analyses examine (i) differentially expressed genes (DEGs) between Hangzhou and Gaizhou populations under control condition, (ii) DEGs as part of a general response in *R*. *oxycephalus* under HTT (iii) temperature responding genes (TRGs) after HTT in Hangzhou and Gaizhou populations.

### Enrichment analysis of differentially expressed genes

GO enrichment of the DEGs was analyzed using GOseq R packages based on Wallenius non-central hyper-geometric distribution, which can adjust for gene length bias in DEGs. The smallest *p*-value indicates the highest degree of GO enrichment. KEGG pathway enrichment of the DEGs was tested using KOBAS software^68^. The hypergeometric test was applied in the enrichment analysis. The minimum threshold of statistical significance was fixed at *p* = 0.05.

### Data confirmation by quantitative PCR analysis

To confirm the accuracy of the transcriptome data, eight DEGs were selected to perform quantitative PCR experiments to verify their expression between Hangzhou and Gaizhou population under control and HTT. PCRs were performed with an Applied Biosystems StepOnePlusTM Real-Time System using SYBR Green I Dye with a 25 μl total volume mixture containing 2 μl of cDNA as the template. The thermal cycling profile consisted of an initial denaturation at 95 °C for 5 min followed by 40 cycles of denaturation at 95 °C for 15 s and annealing/extension at 60 °C for 60 s. An additional temperature-ramping step from 95 °C to 65 °C was used to produce the melting curve. β-actin was used as the internal control because the RNA-Seq data indicated that its expression remained largely constant during temperature treatment. Mean values and standard errors were determined based on two biology replicates, and each with three technical replicates. The specific primers for these genes were designed using PRIMER 5, and information on these primers is additionally provided (Supplementary Table S6). It has been reported that there was 0 false negative for 6 non-DEGs in *Lentinula edodes*^72^. Unfortunately, adding the validation of non-DEGs in the present manuscript is impossible since our brain and RNA samples have been degraded after three years’ storage. However, we must mention here that there were possible false negatives too in this study.

### Statistics analysis

Thermal tolerance data sets were analyzed by analysis of covariance (ANCOVA) with the length or mass as covariates. Corrected CT_Max_ and CT_Min_ values differed by no more than 0.1 °C from actual values (*p = *0.67 for length, *p* = 0.57 for mass); therefore, thermal tolerance differences between Hangzhou and Gaizhou populations were statistically compared by means of one-way analysis of variance (ANOVA) with acclimation temperatures (14, 19, 24 and 29 °C) as factors without statistical adjustment for body size. Tukey’s post hoc tests were used to reveal specific differences among different acclimation temperatures (*p* < 0.05). Statistical analyses were performed using SPSS v 16.0 (SPSS 2006). The thermal tolerance zone area was calculated in Excel 2013.

### Data accessibility

The raw transcriptome reads obtained during this study have been deposited at NCBI Short Read Archive (SRA, http://www.ncbi.nlm.nih.gov/sra/) under the bioproject number PRJNA431525.

## Electronic supplementary material

Supplementary Information
