# Supplementary material for: Regional differences in thermal adaptation of a cold-water fish Rhynchocypris oxycephalus revealed by thermal tolerance and transcriptomic responses
Source: Sci Rep. 2018 Aug 3;8:11703. doi: 10.1038/s41598-018-30074-9 (PMC6076256; doi:10.1038/s41598-018-30074-9)

Regional differences in thermal adaptation of a cold-water fish *Rhynchocypris oxycephalus* revealed by thermal tolerance and transcriptomic responses

Dan Yu[1], Zhi Zhang[1], Zhongyuan Shen[1], Chen Zhang[1] and Huanzhang Liu[1,*]

[1] The Key Laboratory of Aquatic Biodiversity and Conservation of Chinese Academy of Sciences, Institute of Hydrobiology, Chinese Academy of Sciences, Wuhan, 430072, P.R. China

*** Correspondence:** Huanzhang Liu, E-mail: hzliu@ihb.ac.cn

Table S1 The statistics and quality of reads throughout all samples in the *Rhynchocypris oxycephlus* transcriptome

Samples	Read Number	Base Number	Mapped Reads	%≥Q30	Mapped Ratio
Hangzhou population_individual_01_at 19	25202782	7533152992	18878762	0.9139	0.7491
Hangzhou population_individual_02_at 19	25259316	7543398424	18745541	0.9158	0.7421
Hangzhou population_individual_03_at 19	25578835	7636893924	18747587	0.9109	0.7329
Hangzhou population_individual_01_at 29	24135979	7202475988	17591967	0.9139	0.7289
Hangzhou population_individual_02_at 29	23637973	7067120130	17747095	0.9133	0.7508
Hangzhou population_individual_03_at 29	24434487	7293145084	18072353	0.9173	0.7396
Gaizhou population_individual_01_at 19	24309990	7262598362	17496969	0.9165	0.7197
Gaizhou population_individual_02_at 19	24959788	7453903716	17995108	0.9136	0.721
Gaizhou population_individual_03_at 19	25662559	7664277210	18525830	0.9165	0.7219
Gaizhou population_individual_01_at 29	24538656	7326695826	18167493	0.9146	0.7404
Gaizhou population_individual_02_at 29	23967054	7160687662	18255947	0.9117	0.7617
Gaizhou population_individual_03_at 29	24740702	7381595502	18406524	0.9132	0.744

Table S2 Temperature responding genes (just for annotated genes) that were upregulated in response to HTT in the Gaizhou population

#GeneID	Nr_annotation	Hangzhou population at control	Hangzhou population at HTT	Fold-Change	Gaizhou population at control	Gaizhou population at HTT	Fold-Change
c14150.graph_c0	PREDICTED: zinc finger and BTB domain-containing protein 16-A-like [Danio rerio]	2.11	1.92	0.91	0.50	2.76	5.49
c15305.graph_c0	PREDICTED: IQ motif and SEC7 domain-containing protein 1 isoform X4 [Danio rerio]	68.36	83.93	1.23	40.07	96.69	2.41
c15544.graph_c0	PREDICTED: ephrin type-A receptor 8 isoform X3 [Danio rerio]	15.63	16.16	1.03	8.14	22.82	2.80
c17372.graph_c0	PREDICTED: triple functional domain protein-like, partial [Notothenia coriiceps]	9.88	12.85	1.30	2.85	11.12	3.90
c28150.graph_c0	PREDICTED: peptidyl-prolyl cis-trans isomerase C-like [Danio rerio]	53.98	57.69	1.07	26.02	67.03	2.58
c28217.graph_c0	PREDICTED: kinesin-1 heavy chain-like [Gavia stellata]	4.62	6.46	1.40	1.82	8.48	4.67
c28335.graph_c0	PREDICTED: glutamate receptor 2 isoform X2 [Danio rerio]	33.67	37.88	1.13	11.58	42.98	3.71
c28404.graph_c0	PREDICTED: calcium-activated potassium channel subunit alpha-1-like, partial [Stegastes partitus]	11.47	10.60	0.92	2.22	10.71	4.81
c28472.graph_c0	Na+-K+-ATPase [Oryzias melastigma]	39.54	33.46	0.85	13.00	33.62	2.59
c37839.graph_c0	PREDICTED: ras-related protein Rab-6B-like isoform X7 [Maylandia zebra]	47.02	45.42	0.97	9.86	29.11	2.95
c40619.graph_c0	PREDICTED: transcription activator BRG1-like, partial [Chaetura pelagica]	11.88	14.77	1.24	4.65	16.93	3.64
c45116.graph_c0	RAB5A, member RAS oncogene family, a [Danio rerio]	15.90	27.34	1.72	16.96	43.97	2.59
c45641.graph_c0	PREDICTED: dihydropyrimidinase-related protein 2 [Danio rerio]	21.51	31.68	1.47	13.20	41.71	3.16
c49651.graph_c0	PREDICTED: G protein-activated inward rectifier potassium channel 1 [Danio rerio]	8.41	10.67	1.27	1.26	6.27	4.96
c49806.graph_c0	PREDICTED: uncharacterized protein LOC103399007, partial [Cynoglossus semilaevis]	239.26	154.57	0.65	0.06	2.40	39.24
c53030.graph_c0	PREDICTED: kinesin-1 heavy chain [Stegastes partitus]	27.44	28.13	1.03	18.79	47.40	2.52
c54324.graph_c0	PREDICTED: NADH dehydrogenase [ubiquinone] 1 alpha subcomplex subunit 3-like [Astyanax mexicanus]	95.74	131.52	1.37	66.88	151.20	2.26
c54330.graph_c0	PREDICTED: spidroin-2-like [Columba livia]	5.58	7.63	1.37	18.26	49.51	2.71
c55416.graph_c0	PREDICTED: dystonin-like, partial [Danio rerio]	13.45	14.97	1.11	5.97	15.87	2.66
c56941.graph_c0	unknown [Hypophthalmichthys nobilis]	261.13	370.98	1.42	107.24	273.41	2.55
c58025.graph_c0	NADH dehydrogenase [ubiquinone] 1 alpha subcomplex subunit 2 [Danio rerio]	41.44	39.35	0.95	20.02	55.86	2.79

c59876.graph_c0	PREDICTED: peptidyl-prolyl cis-trans isomerase C-like [Danio rerio]	26.69	24.64	0.92	11.83	31.31	2.65
c60695.graph_c0	Polybromo 1, like [Danio rerio]	11.20	20.52	1.83	2.85	15.69	5.51
c60972.graph_c0	PREDICTED: RUN and SH3 domain-containing protein 1 isoform X3 [Danio rerio]	43.41	61.11	1.41	24.89	61.74	2.48
c61923.graph_c0	PREDICTED: dynamin-2-like [Xiphophorus maculatus]	99.17	87.89	0.89	42.10	138.19	3.28
c63224.graph_c0	PREDICTED: protein piccolo isoform X1 [Danio rerio]	16.50	18.47	1.12	7.05	19.41	2.75
c63332.graph_c0	PREDICTED: nuclear factor 1 X-type isoform X6 [Danio rerio]	46.93	42.67	0.91	16.39	45.26	2.76
c65169.graph_c0	reverse transcriptase/ribonuclease H/putative methyltransferase, partial [Tetraodon nigroviridis]	21.01	26.29	1.25	3.04	17.29	5.68
c65178.graph_c0	PREDICTED: low-density lipoprotein receptor-related protein 1-like isoform X1 [Stegastes partitus]	15.73	17.78	1.13	3.81	13.23	3.48
c65237.graph_c0	plasminogen receptor (KT) [Danio rerio]	7.31	13.40	1.83	2.60	9.18	3.52
c65366.graph_c0	nucleolar protein 14 [Danio rerio]	25.94	37.36	1.44	14.35	31.07	2.17
c65883.graph_c0	novel protein similar to human triple functional domain protein TRIO (PTPRF interacting) [Danio rerio]	13.58	15.09	1.11	7.94	21.07	2.65
c65949.graph_c0	serine/threonine-protein kinase pdik1l [Danio rerio]	5.45	8.28	1.52	2.53	7.60	3.00
c65995.graph_c0	PREDICTED: calsyntenin-2 isoform X1 [Danio rerio]	19.95	21.29	1.07	10.20	22.19	2.18
c66331.graph_c0	PREDICTED: cytochrome b-c1 complex subunit 10-like isoform X1 [Neolamprologus brichardi]	5.37	11.45	2.13	3.05	10.59	3.48
c68056.graph_c1	PREDICTED: uncharacterized protein LOC102083087 [Oreochromis niloticus]	5.76	2.53	0.44	0.14	0.81	5.67
c68056.graph_c2	PREDICTED: uncharacterized protein LOC102083148 [Oreochromis niloticus]	2.91	1.46	0.50	0.08	0.41	5.33
c68458.graph_c0	uncharacterized protein LOC619267 [Danio rerio]	13.41	15.85	1.18	7.71	20.06	2.60
c69321.graph_c0	metabotropic glutamate receptor 3 precursor [Danio rerio]	20.20	21.05	1.04	13.30	29.30	2.20
c69335.graph_c0	TGF-beta receptor type-1 precursor [Danio rerio]	8.70	12.48	1.43	10.51	27.38	2.60
c69485.graph_c0	PREDICTED: triple functional domain protein isoform X3 [Danio rerio]	25.18	29.04	1.15	11.96	26.72	2.23
c70352.graph_c0	PREDICTED: uncharacterized protein LOC101157274 [Oryzias latipes]	26.66	24.10	0.90	12.87	53.25	4.14
c70826.graph_c0	PREDICTED: F-box only protein 33 [Danio rerio]	13.18	22.81	1.73	8.79	24.89	2.83
c71106.graph_c0	PREDICTED: histone-lysine N-methyltransferase NSD3 isoform X2 [Danio rerio]	11.61	12.83	1.10	6.51	14.56	2.24
c71223.graph_c0	PREDICTED: leucine-rich repeat-containing protein 4B-like [Astyanax mexicanus]	8.67	10.64	1.23	6.32	16.14	2.55
c71386.graph_c0	PREDICTED: wiskott-Aldrich syndrome protein family member 3-like [Danio rerio]	29.13	49.54	1.70	20.16	55.11	2.73
c71682.graph_c0	PREDICTED: TBC1 domain family member 9 [Danio rerio]	11.85	14.46	1.22	10.90	24.56	2.25

c71884.graph_c0	rho-related GTP-binding protein RhoU [Danio rerio]	6.03	9.79	1.62	3.03	8.69	2.87
c72032.graph_c0	PREDICTED: potassium voltage-gated channel subfamily F member 1 [Danio rerio]	4.31	8.06	1.87	5.59	13.44	2.41
c72369.graph_c0	PREDICTED: uncharacterized protein LOC103908814 [Danio rerio]	27.81	23.36	0.84	22.16	57.19	2.58
c72394.graph_c1	PREDICTED: myosin-10-like, partial [Fulmarus glacialis]	29.77	34.63	1.16	13.93	31.57	2.27
c72528.graph_c0	signal transducer and activator of transcription 5b [Ctenopharyngodon idella]	5.77	10.01	1.73	4.53	10.35	2.29
c72685.graph_c0	Zgc:123049 [Danio rerio]	8.78	9.40	1.07	5.04	14.47	2.87
c72838.graph_c0	uncharacterized protein LOC767635 [Danio rerio]	10.43	10.81	1.04	1.21	7.08	5.84
c73002.graph_c0	metabotropic glutamate receptor 5a [Danio rerio]	27.81	40.59	1.46	14.47	36.15	2.50
c73358.graph_c0	solute carrier family 34 (type II sodium/phosphate cotransporter), member 2a [Danio rerio]	1.07	3.17	2.97	0.17	1.41	8.24
c73422.graph_c0	PREDICTED: small G protein signaling modulator 1-like [Danio rerio]	22.10	28.38	1.28	10.36	24.37	2.35
c73845.graph_c0	BDNF/NT-3 growth factors receptor precursor [Danio rerio]	18.78	21.86	1.16	10.66	24.77	2.32
c73923.graph_c0	PREDICTED: metabotropic glutamate receptor 5-like [Danio rerio]	7.46	7.76	1.04	5.01	11.44	2.29
c74006.graph_c0	PREDICTED: ryanodine receptor 2-like [Lepisosteus oculatus]	22.29	26.49	1.19	13.52	34.52	2.55
c74014.graph_c0	PREDICTED: carcinoembryonic antigen-related cell adhesion molecule 2-like [Danio rerio]	1.82	2.70	1.49	0.37	2.30	6.14
c74245.graph_c0	--	78.83	36.52	0.46	77.03	178.74	2.32
c74264.graph_c0	metabotropic glutamate receptor 5-like precursor [Danio rerio]	17.76	21.72	1.22	7.56	23.97	3.17
c75074.graph_c0	Smarca2 protein, partial [Danio rerio]	24.96	43.89	1.76	12.70	36.58	2.88
c76116.graph_c0	PREDICTED: unconventional myosin-IXa-like isoform X2 [Astyanax mexicanus]	16.30	17.69	1.08	9.17	19.73	2.15
c77330.graph_c0	PREDICTED: uncharacterized protein LOC101884834 [Danio rerio]	19.96	16.23	0.81	9.00	36.93	4.10
c77368.graph_c0	PREDICTED: protocadherin alpha-8-like isoform X2 [Danio rerio]	2.80	2.16	0.77	1.03	4.27	4.13
c77401.graph_c0	PREDICTED: uncharacterized protein LOC103908814 [Danio rerio]	7.76	6.27	0.81	23.27	91.85	3.95
c77475.graph_c0	PREDICTED: kelch-like protein 22 [Danio rerio]	19.38	20.07	1.04	3.61	8.74	2.42
c77511.graph_c0	ataxin-1 [Danio rerio]	5.29	10.19	1.93	2.44	10.78	4.42
c77795.graph_c0	PREDICTED: small G protein signaling modulator 1-like [Danio rerio]	18.59	25.78	1.39	10.79	23.64	2.19
c78230.graph_c0	PREDICTED: coiled-coil domain-containing protein 107 isoform X2 [Danio rerio]	14.85	17.69	1.19	9.45	22.93	2.43
c78546.graph_c0	PREDICTED: uncharacterized protein LOC101170903, partial [Oryzias latipes]	1.70	3.82	2.25	0.17	3.19	18.28

c78909.graph_c0	carbonyl reductase [NADPH] 1 [Danio rerio]	13.68	20.15	1.47	11.42	25.45	2.23
c78978.graph_c0	60S ribosomal protein L38, partial [Oryzias melastigma]	97.39	117.08	1.20	41.12	102.21	2.49
c79364.graph_c0	PREDICTED: uncharacterized protein LOC103045776 [Astyanax mexicanus]	4.47	8.68	1.94	5.24	13.01	2.49
c79860.graph_c1	Zgc:110695 protein [Danio rerio]	20.46	41.28	2.02	21.18	47.56	2.25
c81006.graph_c0	PREDICTED: tomoregulin-1 isoform X1 [Danio rerio]	12.42	20.46	1.65	6.30	14.02	2.23
c81148.graph_c1	PREDICTED: uncharacterized protein LOC105014162 isoform X1 [Esox lucius]	1.17	1.81	1.55	0.86	7.07	8.19
c81352.graph_c0	PREDICTED: uncharacterized protein LOC104948991, partial [Notothenia coriiceps]	5.98	6.77	1.13	4.94	13.74	2.78
c81965.graph_c0	solute carrier family 25 member 47-A [Danio rerio]	4.30	11.63	2.70	3.76	10.07	2.68
c82221.graph_c1	PREDICTED: histone H3-like [Cynoglossus semilaevis]	12.73	27.09	2.13	9.92	32.50	3.28
c82311.graph_c0	TAK1-binding protein 1 [Ctenopharyngodon idella]	11.88	15.84	1.33	6.85	16.67	2.43
c82450.graph_c0	ORF2-encoded protein [Danio rerio]	1.11	1.13	1.02	1.58	7.02	4.45
c82765.graph_c0	PREDICTED: transmembrane protein 117 isoform X1 [Astyanax mexicanus]	5.47	11.04	2.02	3.49	8.92	2.56
c82963.graph_c1	PREDICTED: uncharacterized protein C14orf37 homolog isoform X3 [Astyanax mexicanus]	89.38	105.78	1.18	38.85	85.15	2.19
c82980.graph_c1	PREDICTED: rho GTPase-activating protein 12-like isoform X4 [Astyanax mexicanus]	7.61	14.02	1.84	8.14	20.79	2.55
c82991.graph_c0	complement C3-H1 [Cyprinus carpio]	1.93	1.64	0.85	0.08	1.09	13.31
c83429.graph_c0	PREDICTED: eukaryotic translation initiation factor 2-alpha kinase 4 [Danio rerio]	9.56	11.39	1.19	9.03	20.96	2.32
c83430.graph_c0	Zgc:171298 protein [Danio rerio]	16.57	25.90	1.56	9.99	22.06	2.21
c83681.graph_c0	transferrin receptor protein 2 [Danio rerio]	1.65	2.74	1.66	1.91	6.17	3.22
c84582.graph_c0	PREDICTED: zinc finger protein 341 isoform X1 [Danio rerio]	4.62	7.62	1.65	3.58	8.74	2.44
c84787.graph_c0	PREDICTED: testosterone 17-beta-dehydrogenase 3 isoform X2 [Danio rerio]	1.78	3.90	2.19	1.82	7.78	4.28
c84977.graph_c0	LOC100149441 protein [Danio rerio]	3.89	3.48	0.89	0.94	4.25	4.50
c85350.graph_c0	uncharacterized protein LOC560084 [Danio rerio]	3.17	5.64	1.78	0.88	3.68	4.17
c85391.graph_c0	PREDICTED: VPS10 domain-containing receptor SorCS3 [Oreochromis niloticus]	4.96	5.95	1.20	2.61	6.12	2.35
c85426.graph_c0	PREDICTED: CUGBP Elav-like family member 5-like isoform X1 [Pundamilia nyererei]	15.10	16.07	1.06	9.36	22.73	2.43
c85707.graph_c0	PREDICTED: CUGBP Elav-like family member 4 isoform X2 [Cynoglossus semilaevis]	7.53	8.15	1.08	3.86	8.75	2.27
c85894.graph_c0	PREDICTED: serine/threonine-protein kinase Nek3-like isoform X1 [Astyanax mexicanus]	57.38	76.40	1.33	39.72	113.25	2.85

c86036.graph_c0	PREDICTED: homeobox protein vent1-like [Larimichthys crocea]	0.96	1.65	1.71	1.07	4.58	4.29
c86155.graph_c0	PREDICTED: solute carrier family 12 member 5 isoform X2 [Danio rerio]	73.09	93.33	1.28	31.46	89.91	2.86
c86194.graph_c0	PREDICTED: kelch-like protein 35 [Danio rerio]	0.97	1.90	1.97	1.48	4.77	3.23
c86225.graph_c0	PREDICTED: RNA-binding protein 7 isoform X1 [Danio rerio]	19.49	27.94	1.43	7.58	25.08	3.31
c86280.graph_c0	PREDICTED: 3-oxoacyl-[acyl-carrier-protein] synthase, mitochondrial [Danio rerio]	8.50	12.22	1.44	4.85	11.60	2.39
c86333.graph_c0	PREDICTED: period 1 isoform X1 [Danio rerio]	4.56	4.23	0.93	1.28	3.51	2.74
c86726.graph_c1	metal response element-binding transcription factor-1 [Cyprinus carpio]	5.87	10.12	1.72	3.63	11.82	3.25
c86808.graph_c1	dual specificity protein phosphatase 16 [Danio rerio]	5.98	16.86	2.82	5.27	18.69	3.54
c86915.graph_c0	potassium voltage-gated channel subfamily A member 6 [Danio rerio]	1.56	3.97	2.55	1.61	5.68	3.52
c87043.graph_c1	histone H1, partial [Astyanax paranae]	4.58	14.29	3.12	3.31	9.69	2.93
c87223.graph_c0	PREDICTED: ryanodine receptor 2 [Danio rerio]	24.28	31.71	1.31	15.28	39.62	2.59
c87223.graph_c1	PREDICTED: ryanodine receptor 2 [Danio rerio]	27.09	35.72	1.32	20.53	45.45	2.21
c87630.graph_c1	PREDICTED: uncharacterized protein LOC791177 isoform X2 [Danio rerio]	30.67	52.35	1.71	20.43	45.11	2.21
c87663.graph_c0	PREDICTED: AF4/FMR2 family member 2 [Danio rerio]	3.14	3.02	0.96	2.01	4.85	2.41
c87778.graph_c0	unnamed protein product [Oncorhynchus mykiss]	9.73	10.00	1.03	2.83	8.86	3.13
c87860.graph_c1	zinc finger and BTB domain-containing protein 20 [Danio rerio]	4.31	3.78	0.88	2.62	6.66	2.54
c88327.graph_c0	PREDICTED: beta-adducin isoform X2 [Danio rerio]	48.41	59.74	1.23	37.36	89.94	2.41
c88366.graph_c1	SH3-domain GRB2-like 2-like [Danio rerio]	35.86	61.12	1.70	29.21	76.92	2.63
c88452.graph_c0	PREDICTED: zinc finger BED domain-containing protein 1-like isoform X1 [Stegastes partitus]	5.84	9.57	1.64	0.53	7.89	14.86
c88745.graph_c1	PREDICTED: uncharacterized protein LOC103139521 [Poecilia formosa]	1.34	0.79	0.59	0.11	7.74	72.23
c89024.graph_c1	PREDICTED: chloride channel protein 1 [Danio rerio]	10.01	17.67	1.77	6.09	13.16	2.16
c89194.graph_c0	MAM and LDL-receptor class A domain-containing protein 1 precursor [Danio rerio]	0.49	1.26	2.57	0.96	6.56	6.80
c89377.graph_c1	PREDICTED: protocadherin alpha-8-like isoform X1 [Danio rerio]	1.53	1.09	0.71	0.53	2.14	4.05
c89429.graph_c1	PREDICTED: uncharacterized protein LOC103911222, partial [Danio rerio]	75.28	100.69	1.34	25.82	63.41	2.46
c89648.graph_c0	PREDICTED: TBC1 domain family member 31 [Danio rerio]	3.93	4.78	1.22	4.45	11.85	2.66
c89751.graph_c0	PREDICTED: NMDA receptor-regulated protein 2 isoform X1 [Danio rerio]	4.63	6.87	1.48	3.34	8.58	2.57

c89789.graph_c0	inositol-3-phosphate synthase [Cyprinus carpio]	51.30	157.54	3.07	36.77	149.58	4.07
c90314.graph_c0	kinesin-like protein KIF20A [Danio rerio]	0.70	1.40	2.00	0.55	2.68	4.91
c90583.graph_c0	PREDICTED: trichohyalin-like [Danio rerio]	17.23	23.77	1.38	10.21	29.13	2.85
c90762.graph_c0	PREDICTED: TGF-beta receptor type-2 [Danio rerio]	4.35	11.74	2.70	3.78	10.76	2.85
c90918.graph_c0	transient receptor potential cation channel subfamily c member 6b, partial [Danio rerio]	5.99	5.37	0.90	4.67	15.87	3.40
c90923.graph_c0	PREDICTED: uncharacterized protein LOC102078155 [Oreochromis niloticus]	1.50	1.55	1.03	0.34	4.37	12.78
c90959.graph_c0	PREDICTED: L-rhamnose-binding lectin CSL3 [Danio rerio]	19.75	53.25	2.70	0.25	2.43	9.84
c91079.graph_c0	ryanodine receptor 1 [Danio rerio]	5.75	9.16	1.59	4.79	16.48	3.44
c91333.graph_c0	leptin receptor long form [Ctenopharyngodon idella]	1.53	2.47	1.61	1.38	4.01	2.90
c91460.graph_c0	Pol polyprotein [Dicentrarchus labrax]	1.56	2.79	1.78	1.49	6.37	4.28
c91520.graph_c1	PREDICTED: spectrin beta chain, brain 1 isoform X5 [Danio rerio]	29.36	38.99	1.33	17.19	37.84	2.20
c91560.graph_c0	appa [Danio rerio]	174.52	218.47	1.25	79.46	176.84	2.23
c91606.graph_c1	insulin receptor substrate 2 [Danio rerio]	4.92	7.11	1.45	2.94	7.25	2.47
c91750.graph_c0	PREDICTED: zinc finger BED domain-containing protein 1-like [Danio rerio]	1.30	0.47	0.36	0.34	3.42	9.94
c91814.graph_c0	uncharacterized protein LOC368754 [Danio rerio]	0.83	1.81	2.17	1.05	3.51	3.34
c91883.graph_c1	PREDICTED: kelch-like protein 24-like [Astyanax mexicanus]	8.64	10.80	1.25	5.95	14.15	2.38
c92138.graph_c1	E3 ubiquitin-protein ligase NEURL1 [Danio rerio]	7.82	10.39	1.33	4.90	11.25	2.30
c92238.graph_c0	PREDICTED: receptor-transporting protein 2 [Danio rerio]	0.80	0.53	0.66	0.97	3.76	3.89
c92256.graph_c0	PREDICTED: neural cell adhesion molecule 1-like [Danio rerio]	12.90	26.03	2.02	8.27	20.67	2.50
c92461.graph_c0	PREDICTED: dyslexia-associated protein KIAA0319-like isoform X2 [Danio rerio]	8.72	11.85	1.36	6.17	13.86	2.25
c92589.graph_c1	PREDICTED: RNA-directed DNA polymerase from mobile element jockey-like [Oreochromis niloticus]	0.90	0.28	0.31	0.84	2.71	3.21
c92630.graph_c0	unnamed protein product [Oncorhynchus mykiss]	2.06	2.96	1.44	1.10	6.56	5.99
c92894.graph_c0	tubulointerstitial nephritis antigen-like precursor [Danio rerio]	45.18	57.91	1.28	20.71	47.99	2.32
c93024.graph_c0	PREDICTED: rhophilin-1 isoform X1 [Danio rerio]	9.97	14.52	1.46	5.06	13.37	2.64
c93048.graph_c0	MAM and LDL-receptor class A domain-containing protein 1 precursor [Danio rerio]	2.04	2.45	1.20	3.32	25.68	7.74
c93371.graph_c1	PREDICTED: BAG family molecular chaperone regulator 5 isoform X1 [Danio rerio]	9.34	17.13	1.83	6.47	15.39	2.38

c93609.graph_c0	ryanodine receptor 1 [Danio rerio]	9.26	12.48	1.35	9.11	20.46	2.25
c93621.graph_c0	PREDICTED: unconventional myosin-VIIb-like [Danio rerio]	1.66	3.33	2.00	0.72	3.19	4.45
c93664.graph_c0	PREDICTED: uncharacterized protein LOC100003811 [Danio rerio]	4.23	8.01	1.89	0.92	2.72	2.96
c93693.graph_c0	hamartin [Danio rerio]	15.20	29.65	1.95	8.62	22.49	2.61
c93851.graph_c2	PREDICTED: CREB3 regulatory factor isoform X2 [Danio rerio]	14.12	16.99	1.20	6.81	15.59	2.29
c93916.graph_c1	receptor-type tyrosine-protein phosphatase mu precursor [Danio rerio]	13.33	17.27	1.29	7.97	17.09	2.14
c93940.graph_c0	abhydrolase domain-containing protein 2-A [Danio rerio]	19.76	29.57	1.50	11.56	24.99	2.16
c94020.graph_c0	PREDICTED: neurocan core protein [Danio rerio]	8.64	13.08	1.51	6.17	15.26	2.47
c94023.graph_c0	reverse transcriptase [Oryzias latipes]	0.33	0.22	0.67	0.75	2.68	3.58
c94104.graph_c0	ReO_6 [Oryzias latipes]	2.08	2.19	1.05	1.18	5.50	4.68
c94153.graph_c0	PREDICTED: Kv channel-interacting protein 1 isoform X2 [Poecilia reticulata]	44.24	52.03	1.18	23.54	52.09	2.21
c94169.graph_c0	Zgc:158664 protein [Danio rerio]	23.58	39.16	1.66	7.17	18.01	2.51
c94478.graph_c0	PREDICTED: uncharacterized protein LOC103911013 [Danio rerio]	0.52	0.26	0.50	0.30	2.36	7.98
c94575.graph_c1	Vat1 protein, partial [Danio rerio]	88.05	93.10	1.06	53.15	114.88	2.16
c94678.graph_c0	PREDICTED: uncharacterized protein LOC101886116 [Danio rerio]	4.47	4.53	1.01	1.13	3.91	3.45
c94711.graph_c0	PREDICTED: stereocilin-like [Danio rerio]	1.95	3.41	1.75	1.52	3.66	2.40
c94815.graph_c0	PREDICTED: uncharacterized protein LOC556776 [Danio rerio]	8.32	11.74	1.41	1.63	5.56	3.42
c94845.graph_c0	PREDICTED: oxygen-regulated protein 1 [Danio rerio]	0.60	1.72	2.88	0.17	1.90	10.89
c94917.graph_c0	pol polyprotein, partial [Takifugu rubripes]	29.53	32.32	1.09	13.38	31.73	2.37
c94957.graph_c0	cytoplasmic dynein 1 heavy chain 1 [Danio rerio]	63.68	90.53	1.42	26.93	66.35	2.46
c94967.graph_c0	PREDICTED: uncharacterized protein LOC102078893 [Oreochromis niloticus]	0.76	0.90	1.18	0.09	1.00	10.70
c95091.graph_c0	PREDICTED: uncharacterized protein LOC436616 [Danio rerio]	20.86	20.12	0.96	13.63	42.17	3.10
c95129.graph_c0	PREDICTED: polybromo 1, like isoform X1 [Danio rerio]	14.56	24.50	1.68	8.21	22.81	2.78
c95139.graph_c0	metal response element-binding transcription factor-1 [Cyprinus carpio]	7.24	13.44	1.86	3.97	14.45	3.64
c95178.graph_c0	PREDICTED: uncharacterized protein LOC101884127 [Danio rerio]	3.15	8.71	2.76	3.65	20.66	5.66
c95285.graph_c0	PREDICTED: uncharacterized protein LOC795173 [Danio rerio]	272.21	249.74	0.92	0.13	2.77	21.14

c95357.graph_c0	PREDICTED: cullin-3-like [Neolamprologus brichardi]	22.86	23.40	1.02	6.72	24.76	3.68
c20889.graph_c0	PREDICTED: uncharacterized protein LOC102307237 [Haplochromis burtoni]	0.05	0.02	0.43	0.20	0.95	4.74
c60781.graph_c0	PREDICTED: uncharacterized protein si:ch211-132g1.3 isoform X6 [Danio rerio]	0.73	0.00	0.00	3.15	14.68	4.66
c61942.graph_c0	PREDICTED: uncharacterized protein LOC100003687 [Danio rerio]	0.13	0.05	0.42	0.47	4.19	8.95
c70330.graph_c0	PREDICTED: otogelin-like protein [Danio rerio]	0.16	0.26	1.65	0.49	2.38	4.83
c71542.graph_c0	PREDICTED: RNA-directed DNA polymerase from mobile element jockey-like [Haplochromis burtoni]	0.00	0.14	--	0.15	0.91	6.29
c72061.graph_c0	PREDICTED: uncharacterized protein LOC101159255 [Oryzias latipes]	0.30	0.28	0.94	0.06	1.08	17.89
c72873.graph_c0	protein FAM151A [Danio rerio]	0.06	0.07	1.05	0.21	2.43	11.85
c75895.graph_c0	gag-like protein [Danio rerio]	0.52	0.23	0.45	0.23	1.70	7.25
c77306.graph_c1	PREDICTED: piggyBac transposable element-derived protein 4-like [Larimichthys crocea]	0.60	0.48	0.81	1.26	6.29	5.01
c77635.graph_c0	reverse transcriptase, partial [Clarias batrachus]	0.11	0.11	1.00	1.07	4.58	4.27
c78438.graph_c0	indian hedgehog B protein precursor [Danio rerio]	0.09	0.20	2.27	0.21	1.97	9.19
c80101.graph_c0	unnamed protein product, partial [Oncorhynchus mykiss]	0.31	1.62	5.23	0.25	1.38	5.62
c81203.graph_c0	PREDICTED: alpha-(1,3)-fucosyltransferase 4-like [Lepisosteus oculatus]	0.29	0.57	2.00	0.16	1.49	9.27
c83205.graph_c0	PREDICTED: NACHT, LRR and PYD domains-containing protein 12 [Danio rerio]	0.00	0.25	--	0.63	2.69	4.29
c85388.graph_c0	solute carrier family 1 (glutamate transporter), member 8b [Danio rerio]	0.16	0.14	0.88	0.12	1.77	14.15
c86226.graph_c0	TPA: putative transposase [Xenopus (Silurana) tropicalis]	0.71	0.42	0.60	0.96	3.76	3.92
c87660.graph_c0	FFA-1 protein [Xenopus laevis]	0.01	0.23	17.00	0.78	8.17	10.52
c89523.graph_c0	unnamed protein product [Oncorhynchus mykiss]	0.90	2.14	2.39	0.39	3.92	10.01
c89532.graph_c0	PREDICTED: uncharacterized protein LOC103397171 [Cynoglossus semilaevis]	0.24	0.50	2.10	0.70	4.10	5.88
c91491.graph_c0	unnamed protein product [Tetraodon nigroviridis]	0.28	0.21	0.74	0.22	2.88	13.30
c93139.graph_c0	PREDICTED: uncharacterized protein LOC101882576 [Danio rerio]	0.17	0.52	3.06	1.09	6.04	5.53

Table S3 Temperature responding genes (just for annotated genes) that were downregulated in response to HTT in the Gaizhou population

#GeneID	Nr_annotation	Hangzhou population at control	Hangzhou population at HTT	Fold-Change	Gaizhou population at control	Gaizhou population at HTT	Fold-Change
c27955.graph_c0	cyclin-dependent kinase 1 [Danio rerio]	1.78	1.06	0.60	4.35	0.61	0.14
c28419.graph_c0	3-beta-hydroxysteroid-Delta(8),Delta(7)-isomerase [Danio rerio]	3.70	2.47	0.67	10.08	2.37	0.24
c40735.graph_c0	fatty acid binding protein 7, brain, a [Danio rerio]	577.74	380.89	0.66	620.01	322.70	0.52
c41023.graph_c0	hemoglobin beta chain [Hypophthalmichthys molitrix]	33.21	15.62	0.47	191.75	13.58	0.07
c59557.graph_c0	PREDICTED: sterol-4-alpha-carboxylate 3-dehydrogenase, decarboxylating isoform X2 [Danio rerio]	3.94	1.44	0.37	5.36	0.69	0.13
c61269.graph_c0	protein S100-B [Danio rerio]	493.94	342.44	0.69	840.28	440.48	0.52
c62940.graph_c0	solute carrier family 13, member 5 [Danio rerio]	2.57	1.39	0.54	1.46	0.24	0.16
c65862.graph_c0	thyroid hormone receptor beta [Rana temporaria]	2.42	1.00	0.41	3.95	1.28	0.32
c66347.graph_c0	3-keto-steroid reductase [Danio rerio]	2.23	1.15	0.52	6.35	2.21	0.35
c67390.graph_c0	PREDICTED: uncharacterized protein LOC101884517 [Danio rerio]	1.01	2.37	2.35	1.29	0.34	0.27
c67980.graph_c0	tubulin, alpha 2 [Danio rerio]	55.51	35.77	0.64	50.68	24.08	0.48
c68329.graph_c0	uncharacterized protein LOC555377 [Danio rerio]	5.80	3.16	0.55	10.01	3.27	0.33
c68615.graph_c0	CD82 molecule b [Danio rerio]	13.25	7.28	0.55	11.18	5.47	0.49
c68843.graph_c0	PREDICTED: uncharacterized protein LOC100537439 [Danio rerio]	2.52	0.64	0.26	6.87	2.06	0.30
c71844.graph_c0	solute carrier family 22 member 2 [Danio rerio]	4.86	3.28	0.68	3.85	1.38	0.36
c72536.graph_c0	DNA replication licensing factor MCM3 [Danio rerio]	2.04	1.53	0.75	2.38	0.83	0.35
c73049.graph_c0	vestigial like 4 like [Danio rerio]	2.08	1.08	0.52	1.01	0.25	0.25
c73264.graph_c0	PREDICTED: apolipoprotein L3 isoform X3 [Danio rerio]	4.59	6.57	1.43	3.22	0.73	0.23
c73295.graph_c0	PREDICTED: bone morphogenetic protein 3B [Danio rerio]	4.93	3.37	0.69	6.95	3.39	0.49
c73300.graph_c0	PREDICTED: isopentenyl-diphosphate Delta-isomerase 1 isoform X1 [Danio rerio]	19.66	12.13	0.62	35.69	12.53	0.35
c73377.graph_c0	endothelial lipase precursor [Danio rerio]	5.96	3.02	0.51	9.09	3.28	0.36
c73640.graph_c0	type II iodothyronine deiodinase [Danio rerio]	6.91	7.31	1.06	13.28	4.97	0.37

c73653.graph_c0	PREDICTED: tumor protein p53-inducible nuclear protein 2-like [Danio rerio]	15.66	8.65	0.55	24.38	12.73	0.52
c73983.graph_c0	elongation of very long chain fatty acids protein 7 [Ictalurus furcatus]	2.44	1.46	0.60	5.41	1.85	0.34
c74172.graph_c0	PREDICTED: retinoic acid receptor responder protein 3-like isoform X1 [Astyanax mexicanus]	23.45	14.49	0.62	30.76	12.25	0.40
c74387.graph_c0	methylsterol monooxygenase 1 [Danio rerio]	15.77	10.25	0.65	65.77	17.68	0.27
c75144.graph_c0	PREDICTED: traf2 and NCK-interacting protein kinase-like, partial [Leptosomus discolor]	12.88	7.07	0.55	17.21	7.97	0.46
c75231.graph_c0	PREDICTED: polypyrimidine tract-binding protein 2 isoform X1 [Danio rerio]	12.17	7.96	0.65	15.00	8.03	0.54
c75398.graph_c0	cyclin A2 [Carassius auratus]	1.84	2.02	1.10	3.74	1.02	0.27
c76218.graph_c0	lanosterol 14-alpha demethylase [Danio rerio]	21.04	10.09	0.48	30.79	10.12	0.33
c77460.graph_c0	PREDICTED: nitrogen fixation cluster-like isoform X1 [Danio rerio]	20.23	10.94	0.54	11.92	6.04	0.51
c77582.graph_c0	dihydropyridine receptor beta 1a subunit [Danio rerio]	1.40	0.37	0.26	1.47	0.38	0.26
c77938.graph_c0	PREDICTED: selenium-binding protein 1 isoform X1 [Danio rerio]	7.34	7.11	0.97	6.87	3.62	0.53
c78147.graph_c0	cyclin-L1 [Danio rerio]	13.84	10.17	0.73	20.39	9.85	0.48
c78500.graph_c0	PREDICTED: uncharacterized protein LOC103908834 [Danio rerio]	1.53	1.98	1.29	1.24	0.32	0.26
c78565.graph_c0	PREDICTED: receptor-type tyrosine-protein kinase FLT3 [Danio rerio]	1.74	3.18	1.82	2.80	0.75	0.27
c78768.graph_c0	macrophage expressed 1, tandem duplicate 1 precursor [Danio rerio]	2.41	3.40	1.41	1.35	0.38	0.28
c78900.graph_c0	PREDICTED: G patch domain-containing protein 8 isoform X3 [Danio rerio]	65.39	44.79	0.68	121.00	59.11	0.49
c79039.graph_c0	PREDICTED: SH3 domain-binding glutamic acid-rich-like protein [Astyanax mexicanus]	9.83	6.99	0.71	7.21	3.05	0.42
c79142.graph_c0	tetratricopeptide repeat protein 14 [Danio rerio]	33.66	19.90	0.59	46.44	23.00	0.50
c79341.graph_c0	nicotinamide riboside kinase 2 [Danio rerio]	2.54	1.21	0.48	4.28	0.70	0.16
c79447.graph_c0	PREDICTED: hydroxymethylglutaryl-CoA synthase, cytoplasmic isoform X1 [Danio rerio]	20.02	6.13	0.31	52.18	6.89	0.13
c79671.graph_c1	PREDICTED: MAGUK p55 subfamily member 3-like isoform X5 [Danio rerio]	46.00	25.58	0.56	18.29	7.67	0.42
c80469.graph_c0	Unknown (protein for IMAGE:9039285) [Danio rerio]	6.94	3.69	0.53	10.82	5.40	0.50
c80563.graph_c0	tissue factor precursor [Danio rerio]	15.60	10.04	0.64	21.85	7.94	0.36
c80817.graph_c0	PREDICTED: transforming acidic coiled-coil-containing protein 3 isoform X1 [Danio rerio]	0.96	0.89	0.92	1.90	0.59	0.31
c80839.graph_c0	PREDICTED: intersectin 2a isoform X1 [Danio rerio]	6.48	4.12	0.64	8.61	4.41	0.51
c81085.graph_c0	phosphoinositide-3-kinase, regulatory subunit 3a (gamma) [Danio rerio]	3.40	3.52	1.03	9.47	3.76	0.40

c81231.graph_c0	PREDICTED: peptidyl-prolyl cis-trans isomerase FKBP5 isoform X1 [Danio rerio]	18.29	31.56	1.73	82.35	25.63	0.31
c81317.graph_c0	D-3-phosphoglycerate dehydrogenase [Danio rerio]	23.47	16.02	0.68	30.45	15.03	0.49
c81452.graph_c0	Col15a1 protein [Danio rerio]	2.18	2.67	1.22	2.19	0.75	0.34
c81748.graph_c0	PREDICTED: transmembrane channel-like protein 1 [Danio rerio]	0.96	2.19	2.29	1.68	0.41	0.24
c82050.graph_c0	mitochondrial brown fat uncoupling protein 1 [Danio rerio]	35.80	24.00	0.67	37.17	19.14	0.51
c82264.graph_c0	PREDICTED: uracil nucleotide/cysteinyl leukotriene receptor [Danio rerio]	8.97	5.54	0.62	13.24	6.74	0.51
c82374.graph_c0	PREDICTED: electrogenic sodium bicarbonate cotransporter 1-like [Xiphophorus maculatus]	83.93	44.33	0.53	101.45	46.22	0.46
c82425.graph_c0	microtubule-associated protein RP/EB family member 1 [Danio rerio]	24.33	18.56	0.76	31.63	16.45	0.52
c82621.graph_c0	PREDICTED: 1-phosphatidylinositol 4,5-bisphosphate phosphodiesterase beta-2-like, partial [Danio rerio]	4.73	3.06	0.65	6.12	1.52	0.25
c82852.graph_c0	PREDICTED: monocarboxylate transporter 7 [Danio rerio]	4.97	3.02	0.61	4.52	2.23	0.49
c82878.graph_c0	PREDICTED: uncharacterized protein LOC103037945 [Astyanax mexicanus]	1.00	1.63	1.64	0.67	0.13	0.20
c83807.graph_c0	secretory carrier membrane protein 2 isoform 1 [Danio rerio]	15.00	10.87	0.72	19.09	9.58	0.50
c83844.graph_c0	PREDICTED: DNA topoisomerase 2-alpha isoform X1 [Danio rerio]	2.06	1.34	0.65	3.19	1.14	0.36
c84233.graph_c1	condensin complex subunit 2 [Danio rerio]	0.79	1.08	1.38	1.26	0.33	0.27
c84462.graph_c0	sodium-dependent lysophosphatidylcholine symporter 1-B [Danio rerio]	10.11	6.10	0.60	13.65	6.31	0.46
c84538.graph_c0	adenylosuccinate synthetase isozyme 2 [Danio rerio]	9.86	5.23	0.53	10.08	5.07	0.50
c84568.graph_c0	PREDICTED: carnitine O-palmitoyltransferase 1, liver isoform isoform X2 [Danio rerio]	3.26	2.24	0.69	3.39	1.35	0.40
c84682.graph_c0	PREDICTED: cytoskeleton-associated protein 2 [Danio rerio]	1.22	0.46	0.38	2.55	0.78	0.31
c84893.graph_c1	pleckstrin homology domain-containing family A member 8 [Danio rerio]	21.69	12.85	0.59	23.44	11.54	0.49
c85018.graph_c0	PREDICTED: PHD finger protein 13 [Danio rerio]	15.25	8.26	0.54	16.38	8.59	0.52
c85232.graph_c0	retinol dehydrogenase 10-A [Danio rerio]	12.59	9.41	0.75	14.01	7.10	0.51
c85494.graph_c0	PREDICTED: kinesin-like protein KIF11 isoform X1 [Danio rerio]	0.88	0.86	0.98	1.46	0.41	0.28
c85692.graph_c0	PREDICTED: lysine-specific demethylase 6B [Danio rerio]	12.18	7.80	0.64	16.66	5.90	0.35
c85706.graph_c0	Rho family GTPase 1 like [Danio rerio]	33.97	24.41	0.72	39.66	21.16	0.53
c85847.graph_c0	PREDICTED: myomesin-2 [Danio rerio]	7.48	5.28	0.71	6.14	3.00	0.49

c86137.graph_c0	voltage-gated sodium channel Nav1.4b [Danio rerio]	11.61	8.58	0.74	8.18	3.85	0.47
c86212.graph_c0	PREDICTED: uncharacterized protein LOC100003350 [Danio rerio]	6.69	1.61	0.24	4.15	1.07	0.26
c86350.graph_c0	MAWD binding protein like [Danio rerio]	1.95	0.63	0.32	23.11	6.04	0.26
c86549.graph_c1	PREDICTED: G protein-coupled receptor kinase 5-like isoform X2 [Danio rerio]	9.46	6.17	0.65	17.81	8.23	0.46
c86581.graph_c0	3-hydroxy-3-methylglutaryl-coenzyme A reductase [Gobiocypris rarus]	16.78	7.51	0.45	40.72	11.17	0.27
c86732.graph_c0	PREDICTED: B-cell CLL/lymphoma 6 member B protein [Danio rerio]	9.69	5.27	0.54	9.03	2.89	0.32
c87359.graph_c0	PREDICTED: diacylglycerol kinase beta-like [Xiphophorus maculatus]	1.85	0.79	0.43	4.51	0.44	0.10
c87534.graph_c0	PREDICTED: arginine and glutamate-rich protein 1 isoform X2 [Notothenia coriiceps]	48.51	29.55	0.61	67.20	33.37	0.50
c87810.graph_c1	farnesyl pyrophosphate synthase [Danio rerio]	10.21	7.44	0.73	27.58	9.46	0.34
c87896.graph_c0	cryptochrome-1 [Danio rerio]	1.43	1.20	0.83	8.62	3.25	0.38
c88136.graph_c0	PREDICTED: proteolipid protein 1b isoform X3 [Danio rerio]	719.95	447.59	0.62	1059.64	513.37	0.48
c88194.graph_c0	PREDICTED: von Willebrand factor A domain-containing protein 7 isoform X2 [Danio rerio]	3.22	1.47	0.46	4.39	1.25	0.28
c88593.graph_c0	Arntl2 protein [Danio rerio]	1.85	1.02	0.55	4.61	1.92	0.42
c88988.graph_c1	PREDICTED: nuclear factor of activated T-cells 5-like isoform X4 [Danio rerio]	6.01	3.80	0.63	6.67	3.18	0.48
c89028.graph_c0	C13orf22 homolog (H. sapiens), like [Danio rerio]	25.09	16.72	0.67	47.60	22.22	0.47
c89238.graph_c1	hypothetical protein N303_03571, partial [Cuculus canorus]	710.40	981.89	1.38	4414.26	735.66	0.17
c89599.graph_c0	PREDICTED: calcium-activated potassium channel subunit alpha-1-like isoform X7 [Neolamprologus brichardi]	35.24	23.02	0.65	50.95	26.76	0.53
c89610.graph_c0	Baz1a protein [Danio rerio]	2.79	2.22	0.80	3.65	1.67	0.46
c89663.graph_c1	PREDICTED: kinesin-like protein KIF17 isoform X1 [Danio rerio]	83.87	59.21	0.71	56.62	26.49	0.47
c89726.graph_c0	uncharacterized protein LOC100037361 [Danio rerio]	2306.87	1447.00	0.63	5905.05	1309.96	0.22
c89726.graph_c1	uncharacterized protein LOC100192222 [Danio rerio]	1179.59	1478.38	1.25	6351.35	1015.41	0.16
c90201.graph_c1	PREDICTED: supervillin isoform X8 [Danio rerio]	15.26	10.66	0.70	13.63	7.08	0.52
c91161.graph_c1	PREDICTED: uncharacterized protein LOC101882617 [Danio rerio]	3.05	4.48	1.47	1.65	0.18	0.11
c91278.graph_c0	PREDICTED: calcium-binding mitochondrial carrier protein SCaMC-2-B isoform X2 [Danio rerio]	5.28	4.16	0.79	4.99	2.48	0.50
c91297.graph_c0	unnamed protein product, partial [Tetraodon nigroviridis]	5.84	4.89	0.84	10.70	5.47	0.51

c91432.graph_c0	reverse transcriptase [Danio rerio]	18.10	10.14	0.56	17.67	9.45	0.53
c91706.graph_c0	PREDICTED: uncharacterized protein LOC104934881 [Larimichthys crocea]	23.50	16.19	0.69	18.23	4.86	0.27
c92264.graph_c1	PREDICTED: interferon-induced protein 44 [Danio rerio]	38.55	22.38	0.58	23.36	10.44	0.45
c92303.graph_c0	DAZ-associated protein 2 [Danio rerio]	95.33	58.24	0.61	95.60	49.11	0.51
c93259.graph_c0	IGF-II mRNA-binding protein 2a [Danio rerio]	5.05	3.28	0.65	6.75	3.18	0.47
c93422.graph_c0	lysosome membrane protein 2 [Danio rerio]	35.35	20.28	0.57	53.38	21.76	0.41
c93943.graph_c0	PREDICTED: dynein assembly factor 3, axonemal-like [Danio rerio]	2.76	3.73	1.35	16.50	5.42	0.33
c94595.graph_c0	PREDICTED: histone-lysine N-methyltransferase, H3 lysine-79 specific isoform X2 [Danio rerio]	7.77	4.31	0.55	9.99	5.23	0.52
c94732.graph_c0	Krueppel-like factor 13 [Danio rerio]	10.56	8.54	0.81	19.27	10.06	0.52
c94739.graph_c1	PREDICTED: cAMP-specific 3',5'-cyclic phosphodiesterase 4C isoform X3 [Danio rerio]	5.97	2.93	0.49	9.33	4.76	0.51
c94770.graph_c1	PREDICTED: lysine-specific demethylase 6B-like isoform X2 [Astyanax mexicanus]	163.59	107.02	0.65	252.89	119.33	0.47
c95006.graph_c1	fatty acid elongase [Cyprinus carpio]	37.83	27.82	0.74	50.01	25.74	0.51
c51277.graph_c0	PREDICTED: uncharacterized protein LOC102078224 [Oreochromis niloticus]	0.05	0.02	0.43	3.05	0.17	0.06
c52225.graph_c0	A disintegrin and metalloproteinase with thrombospondin motifs 9 [Danio rerio]	0.35	0.33	0.94	0.84	0.18	0.22
c60061.graph_c0	PREDICTED: proline-rich protein 5-like [Danio rerio]	0.75	0.42	0.56	1.05	0.22	0.21
c63203.graph_c0	spindle assembly checkpoint protein Mad2 [Danio rerio]	1.54	0.91	0.59	1.68	0.43	0.26
c68588.graph_c0	PREDICTED: interleukin-12 receptor subunit beta-1 isoform X2 [Danio rerio]	0.80	0.43	0.54	1.24	0.31	0.25
c69277.graph_c0	PREDICTED: cell division cycle 7-related protein kinase isoform X1 [Danio rerio]	0.64	0.63	0.99	5.16	0.31	0.06
c73312.graph_c0	PREDICTED: DNA replication factor Cdt1 [Danio rerio]	0.77	0.35	0.45	1.29	0.36	0.28
c75086.graph_c0	PREDICTED: uncharacterized protein LOC103381760 [Cynoglossus semilaevis]	0.45	0.41	0.91	1.23	0.18	0.15
c76574.graph_c0	cysteinyl leukotriene receptor 1 [Danio rerio]	0.47	0.17	0.36	0.91	0.21	0.23
c80417.graph_c0	PREDICTED: ras and EF-hand domain-containing protein [Danio rerio]	0.91	0.79	0.87	0.93	0.23	0.24
c83292.graph_c0	Si:dkeyp-26a9.1 protein [Danio rerio]	0.73	0.44	0.60	1.38	0.37	0.27

Table S4 Temperature responding genes (just for annotated genes) that were upregulated in response to HTT in the Hangzhou population

#GeneID	Nr_annotation	Hangzhou population at control	Hangzhou population at HTT	Fold-Change	Gaizhou population at control	Gaizhou population at HTT	Fold-Change
c57380.graph_c0	protein CMSS1 [Danio rerio]	6.22	14.06	2.26	9.28	13.56	1.46
c60675.graph_c0	connective tissue growth factor a [Megalobrama amblycephala]	2.94	6.63	2.26	2.94	3.81	1.30
c61437.graph_c0	RecName: Full=Red-sensitive opsin-2; AltName: Full=Opsin-1, long-wave-sensitive 2; Short=Opsin LWS-2; AltName: Full=Red cone photoreceptor pigment 2 [Danio rerio]	0.56	2.85	5.09	1.56	6.61	4.22
c62200.graph_c0	cytohesin 4 [Danio rerio]	2.18	6.97	3.19	2.44	3.94	1.62
c63982.graph_c0	PREDICTED: thiosulfate sulfurtransferase/rhodanese-like domain-containing protein 1 [Danio rerio]	4.12	11.64	2.83	5.51	7.69	1.40
c70326.graph_c0	PREDICTED: RNA-binding protein 20 [Danio rerio]	0.96	4.71	4.89	1.09	1.33	1.22
c71289.graph_c1	CEF-10 precursor [Salmo salar]	5.56	13.79	2.48	6.41	10.54	1.64
c73609.graph_c0	PREDICTED: arginine and glutamate-rich protein 1-A-like [Danio rerio]	0.34	1.70	4.98	0.95	1.09	1.14
c74385.graph_c0	lipocalin-type prostaglandin D synthase-like protein [Danio rerio]	23.70	53.63	2.26	27.79	46.06	1.66
c75848.graph_c0	leucine-rich repeat-containing protein 14B [Danio rerio]	1.20	3.39	2.82	1.70	1.29	0.76
c76802.graph_c0	Ephx1 protein, partial [Danio rerio]	0.24	1.95	8.14	1.21	1.05	0.87
c77515.graph_c0	PREDICTED: uncharacterized protein K02A2.6-like [Xenopus (Silurana) tropicalis]	4.42	10.32	2.33	5.61	7.96	1.42
c78651.graph_c0	PREDICTED: tRNA-specific adenosine deaminase 1 isoform X1 [Danio rerio]	1.51	4.29	2.84	1.61	2.71	1.68
c78704.graph_c0	PREDICTED: protein GRINL1A [Danio rerio]	0.79	2.50	3.16	1.34	3.26	2.43
c79611.graph_c0	ras-like protein family member 11A-like [Danio rerio]	0.96	3.39	3.53	1.70	2.91	1.71
c80276.graph_c0	BAG family molecular chaperone regulator 2 [Danio rerio]	2.44	7.50	3.07	2.80	8.14	2.91
c80419.graph_c0	PREDICTED: tyrosine-protein kinase BTK [Danio rerio]	1.14	2.98	2.61	1.56	1.25	0.80
c81302.graph_c0	PREDICTED: cold shock domain-containing protein C2-like [Pundamilia nyererei]	44.73	98.50	2.20	50.56	96.67	1.91
c82797.graph_c0	PREDICTED: natural cytotoxicity triggering receptor 3 [Danio rerio]	0.66	2.79	4.23	1.55	4.14	2.67
c83008.graph_c0	tubulin-folding cofactor B [Danio rerio]	30.40	62.22	2.05	34.24	53.44	1.56
c84244.graph_c0	syntaxin binding protein 1b [Danio rerio]	8.29	21.58	2.60	9.03	22.63	2.51

c86083.graph_c0	PREDICTED: DNA ligase 4 isoform X1 [Danio rerio]	2.18	5.71	2.61	8.96	18.67	2.08
c86184.graph_c0	PREDICTED: interleukin-1 receptor type 1-like [Danio rerio]	0.57	3.17	5.51	0.68	2.46	3.61
c87218.graph_c0	PREDICTED: fibrous sheath CABYR-binding protein-like isoform X2 [Astyanax mexicanus]	6.16	17.01	2.76	15.69	30.41	1.94
c87474.graph_c1	PREDICTED: adenylate kinase 9 isoform X1 [Danio rerio]	1.12	2.62	2.35	1.12	0.62	0.56
c87926.graph_c0	PREDICTED: kinesin-1 heavy chain isoform X1 [Danio rerio]	19.76	42.80	2.17	22.62	36.65	1.62
c88621.graph_c0	fas-activated serine/threonine kinase [Danio rerio]	7.20	15.32	2.13	8.99	14.89	1.66
c90208.graph_c0	TFIIA-alpha and beta-like factor [Danio rerio]	0.38	2.90	7.66	0.78	2.13	2.73
c90641.graph_c0	unnamed protein product [Oncorhynchus mykiss]	0.38	1.80	4.71	0.41	0.59	1.46
c90749.graph_c0	PREDICTED: ferrochelatase, mitochondrial isoform X1 [Danio rerio]	7.11	17.05	2.40	9.54	13.85	1.45
c91142.graph_c0	PREDICTED: EMILIN-2 [Danio rerio]	0.57	2.55	4.45	0.67	1.56	2.33
c91348.graph_c0	PREDICTED: probable E3 ubiquitin-protein ligase HERC4 [Danio rerio]	5.52	13.19	2.39	7.54	10.96	1.45
c91436.graph_c0	PREDICTED: ubiquitin carboxyl-terminal hydrolase 28 [Danio rerio]	1.07	3.02	2.82	1.82	2.73	1.50
c91652.graph_c0	prostaglandin E synthase 3 [Danio rerio]	22.91	48.81	2.13	27.58	45.01	1.63
c91725.graph_c1	PREDICTED: lipoamide acyltransferase component of branched-chain alpha-keto acid dehydrogenase complex, mitochondrial-like [Astyanax mexicanus]	1.54	4.82	3.12	2.41	6.68	2.77
c91730.graph_c0	PREDICTED: collagen alpha-2(XI) chain isoform X3 [Danio rerio]	1.21	3.77	3.12	1.35	2.84	2.11
c92164.graph_c0	PREDICTED: coiled-coil domain-containing protein 157 [Danio rerio]	0.37	2.90	7.79	0.77	2.08	2.72
c92294.graph_c0	PREDICTED: uncharacterized protein LOC569093 isoform X1 [Danio rerio]	1.61	3.86	2.39	2.23	4.14	1.85
c92400.graph_c0	PREDICTED: dnaJ homolog subfamily B member 1 [Esox lucius]	4.00	17.67	4.42	7.54	14.98	1.99
c92430.graph_c1	PREDICTED: ral guanine nucleotide dissociation stimulator-like 1 isoform X2 [Danio rerio]	1.92	4.90	2.55	2.16	3.17	1.46
c93205.graph_c0	PREDICTED: protein FAM188B [Danio rerio]	6.21	15.73	2.53	13.75	19.90	1.45
c93385.graph_c0	heat shock protein 70 [Danio rerio]	11.93	31.66	2.65	12.05	25.16	2.09
c94008.graph_c1	PREDICTED: ATP-dependent DNA helicase Q5 [Danio rerio]	2.80	7.23	2.58	2.85	5.99	2.10
c94066.graph_c1	Mcl1b [Danio rerio]	32.41	127.24	3.93	46.95	93.86	2.00
c94074.graph_c4	PREDICTED: angio-associated migratory cell protein [Stegastes partitus]	9.34	22.23	2.38	13.36	25.44	1.90
c50786.graph_c0	nuclear autoantigen of 14 kDa [Danio rerio]	2.36	6.73	2.85	1.54	2.33	1.51

c53548.graph_c0	uncharacterized protein LOC393586 [Danio rerio]	5.04	15.36	3.05	4.03	8.73	2.16
c59509.graph_c0	unnamed protein product [Tetraodon nigroviridis]	29.57	79.91	2.70	19.55	31.72	1.62
c62645.graph_c0	PREDICTED: period circadian protein homolog 2-like isoform X1 [Astyanax mexicanus]	3.91	11.74	3.01	3.69	6.50	1.76
c69602.graph_c0	nck-associated protein 1-like [Danio rerio]	5.50	29.30	5.32	4.24	3.90	0.92
c71987.graph_c1	protein S100-A14 [Danio rerio]	10.48	23.55	2.25	9.19	14.23	1.55
c72419.graph_c0	uncharacterized protein LOC393586 [Danio rerio]	5.23	13.38	2.56	3.18	7.35	2.31
c72463.graph_c0	PREDICTED: protein Hikeshi [Astyanax mexicanus]	16.18	37.67	2.33	10.66	21.36	2.00
c74688.graph_c0	CD4-like protein [Ctenopharyngodon idella]	0.97	3.12	3.22	0.84	0.25	0.29
c75208.graph_c0	PREDICTED: doublecortin domain-containing protein 2 [Poecilia reticulata]	1.63	4.87	2.99	1.42	2.33	1.64
c76515.graph_c0	PREDICTED: WAS/WASL-interacting protein family member 1 [Danio rerio]	4.31	9.32	2.16	3.81	4.18	1.10
c77164.graph_c0	PREDICTED: vitamin K epoxide reductase complex subunit 1-like protein 1 [Danio rerio]	4.15	9.92	2.39	3.30	4.97	1.51
c77436.graph_c0	deoxyhypusine hydroxylase [Danio rerio]	13.29	27.21	2.05	12.66	18.87	1.49
c77452.graph_c0	PREDICTED: PH and SEC7 domain-containing protein 1-like isoform X4 [Danio rerio]	2.83	8.05	2.85	2.44	1.97	0.81
c77606.graph_c0	hydroxylysine kinase [Danio rerio]	6.22	13.08	2.10	2.12	2.99	1.41
c79117.graph_c0	receptor accessory protein 3a [Danio rerio]	27.72	63.88	2.30	22.48	29.85	1.33
c79244.graph_c0	PREDICTED: myosin-9 isoform X2 [Danio rerio]	1.43	3.43	2.41	1.04	1.95	1.88
c79558.graph_c0	PREDICTED: acetylcholinesterase collagenic tail peptide isoform X2 [Danio rerio]	5.75	13.94	2.43	1.61	3.56	2.22
c80331.graph_c0	fas-activated serine/threonine kinase [Danio rerio]	8.20	21.94	2.68	8.13	16.69	2.05
c82092.graph_c0	PREDICTED: uncharacterized protein C2orf47 homolog, mitochondrial-like isoform X1 [Danio rerio]	4.04	10.60	2.62	3.64	9.20	2.53
c84013.graph_c0	histone H1-like [Danio rerio]	14.22	36.80	2.59	9.15	19.48	2.13
c84748.graph_c0	probable cation-transporting ATPase 13A2 [Danio rerio]	5.94	13.50	2.27	4.80	9.61	2.00
c85091.graph_c0	granulocyte colony stimulating factor receptor [Carassius auratus]	2.52	6.47	2.56	1.40	1.70	1.22
c86677.graph_c0	PREDICTED: cysteine-rich with EGF-like domain protein 1 [Danio rerio]	13.55	29.91	2.21	12.33	25.12	2.04
c86754.graph_c0	PREDICTED: uncharacterized protein LOC102076765 isoform X1 [Oreochromis niloticus]	8.58	21.56	2.51	3.72	7.24	1.94
c86955.graph_c1	PREDICTED: cardiomyopathy associated 5 isoform X1 [Danio rerio]	0.98	2.36	2.40	0.26	0.48	1.84

c87414.graph_c0	PREDICTED: LOW QUALITY PROTEIN: neuroblast differentiation-associated protein AHNAK [Danio rerio]	1.28	5.48	4.30	1.06	3.15	2.98
c88525.graph_c2	PREDICTED: prolyl 4-hydroxylase, alpha polypeptide I b isoform X3 [Danio rerio]	1.61	5.39	3.35	1.22	2.28	1.87
c89172.graph_c0	PREDICTED: inactive dual specificity phosphatase 27 [Danio rerio]	0.53	2.81	5.28	0.30	0.75	2.49
c89191.graph_c0	PREDICTED: uncharacterized protein LOC104949296 isoform X6 [Notothenia coriiceps]	4.81	11.99	2.49	2.21	1.33	0.60
c89631.graph_c0	PREDICTED: unconventional myosin-XVIIIb isoform X3 [Esox lucius]	1.63	3.87	2.37	1.54	2.56	1.66
c91271.graph_c0	PREDICTED: LOW QUALITY PROTEIN: cyclic nucleotide-gated cation channel beta-1-like [Danio rerio]	3.70	8.73	2.36	3.42	5.42	1.59
c91418.graph_c0	PREDICTED: collagen alpha-1(XII) chain-like isoform X1 [Stegastes partitus]	0.84	3.76	4.47	0.60	0.79	1.32
c92413.graph_c0	Heat shock protein 70 [Danio rerio]	53.72	112.04	2.09	50.39	100.69	2.00
c94486.graph_c0	PREDICTED: uncharacterized protein LOC103359624 [Stegastes partitus]	31.58	66.74	2.11	0.70	0.94	1.35
c94817.graph_c0	PREDICTED: interleukin-1 receptor type 1-like [Danio rerio]	10.82	29.39	2.72	6.75	9.99	1.48
c94866.graph_c1	atrial natriuretic peptide receptor 1 precursor [Danio rerio]	13.78	30.25	2.19	5.17	4.94	0.96
c88089.graph_c0	PREDICTED: deformed epidermal autoregulatory factor 1 homolog isoform X1 [Danio rerio]	2.61	5.62	2.15	2.38	5.40	2.27
c92110.graph_c0	cytochrome P450, family 2, subfamily AD, polypeptide 6 [Danio rerio]	1.08	4.31	3.99	0.37	1.48	4.02

Table S5 Temperature responding genes (just for annotated genes) that were downregulated in response to HTT in the Hangzhou population

#GeneID	Nr_annotation	Hangzhou population at control	Hangzhou population at HTT	Fold-Change	Gaizhou population at control	Gaizhou population at HTT	Fold-Change
c27857.graph_c0	ORF2-encoded protein [Danio rerio]	1.76	0.36	0.21	0.62	0.41	0.66
c28414.graph_c0	PREDICTED: cytochrome c oxidase subunit 8A, mitochondrial-like [Danio rerio]	18.63	7.22	0.39	1.15	0.91	0.79
c29072.graph_c0	PREDICTED: protein NLRC3-like [Danio rerio]	6.42	0.51	0.08	0.36	0.73	2.01
c40705.graph_c0	PREDICTED: ubiquitin-associated protein 1-like isoform X1 [Danio rerio]	6.74	1.81	0.27	1.12	0.89	0.79
c54202.graph_c0	relaxin 3a precursor [Danio rerio]	28.84	14.15	0.49	14.19	10.89	0.77
c57759.graph_c0	intestinal fatty acid binding protein 2b [Cyprinus carpio]	9.43	3.96	0.42	4.40	2.98	0.68
c60814.graph_c0	PREDICTED: plexin domain-containing protein 1-like isoform X2 [Astyanax mexicanus]	5.44	1.74	0.32	4.47	3.02	0.68
c60915.graph_c0	ORF2-encoded protein [Danio rerio]	12.60	3.00	0.24	1.69	2.57	1.52
c61018.graph_c0	PREDICTED: uncharacterized protein LOC104922756 [Larimichthys crocea]	2.61	0.39	0.15	0.02	0.37	14.79
c63454.graph_c0	PREDICTED: SLIT-ROBO Rho GTPase-activating protein 1 [Danio rerio]	0.82	0.13	0.15	0.21	0.18	0.86
c66416.graph_c0	myostatin a [Megalobrama amblycephala]	10.87	4.97	0.46	6.71	5.21	0.78
c68078.graph_c0	PREDICTED: regulator of G-protein signaling 9-binding protein isoform X1 [Danio rerio]	1.77	0.38	0.22	0.68	0.34	0.50
c70583.graph_c0	unnamed protein product [Oncorhynchus mykiss]	2.04	0.27	0.13	0.10	0.29	2.81
c72750.graph_c0	catechol O-methyltransferase [Danio rerio]	8.78	3.86	0.44	3.99	3.17	0.79
c74445.graph_c0	PREDICTED: receptor-type tyrosine-protein phosphatase delta isoform X6 [Danio rerio]	19.65	8.95	0.46	16.01	7.82	0.49
c76396.graph_c0	PREDICTED: rho-related BTB domain-containing protein 1 isoform X2 [Danio rerio]	10.12	4.80	0.47	8.94	5.29	0.59
c76840.graph_c0	kelch-like protein 40a [Danio rerio]	3.21	1.25	0.39	1.41	1.45	1.03
c77414.graph_c0	PREDICTED: uncharacterized protein LOC101478033 [Maylandia zebra]	2.72	0.61	0.23	0.35	0.65	1.87
c78037.graph_c0	uncharacterized protein LOC558132 [Danio rerio]	11.28	3.77	0.33	6.88	5.94	0.86
c78692.graph_c0	pol-like protein [Danio rerio]	3.30	1.50	0.46	0.58	0.21	0.36
c78884.graph_c0	PREDICTED: uncharacterized protein LOC102314156 [Haplochromis burtoni]	4.22	1.54	0.37	0.28	0.38	1.39
c78895.graph_c0	PREDICTED: diacylglycerol kinase eta isoform X4 [Danio rerio]	83.33	34.26	0.41	40.98	34.92	0.85

c80244.graph_c0	PREDICTED: disks large homolog 1 isoform X4 [Latimeria chalumnae]	24.44	9.43	0.39	15.96	14.34	0.90
c80263.graph_c0	proteasome beta 11 subunit [Danio rerio]	3.16	0.00	0.00	2.15	0.00	0.00
c80271.graph_c0	spondin-1 precursor [Danio rerio]	13.31	6.42	0.48	7.09	6.61	0.93
c80396.graph_c0	PREDICTED: galaxin-like isoform X2 [Danio rerio]	3.01	0.55	0.18	1.46	1.03	0.71
c80770.graph_c1	PREDICTED: insulin-degrading enzyme-like [Pundamilia nyererei]	1.39	0.36	0.26	0.81	0.38	0.47
c81086.graph_c0	PREDICTED: V-set and transmembrane domain-containing protein 4 [Danio rerio]	1.73	0.49	0.29	1.57	1.52	0.96
c81335.graph_c0	unnamed protein product [Oncorhynchus mykiss]	4.34	1.20	0.28	0.97	1.14	1.17
c81766.graph_c0	PREDICTED: voltage-dependent L-type calcium channel subunit alpha-1D-like, partial [Notothenia coriiceps]	4.75	1.12	0.24	3.05	1.35	0.44
c82190.graph_c0	Proteasome (prosome, macropain) subunit, beta type, 8 [Danio rerio]	6.98	0.26	0.04	5.60	4.27	0.76
c82753.graph_c0	PREDICTED: uncharacterized protein LOC101883064 [Danio rerio]	6.24	2.31	0.37	1.51	0.61	0.40
c82847.graph_c0	PREDICTED: uncharacterized protein LOC105015351 [Esox lucius]	11.82	4.15	0.35	7.63	14.99	1.96
c84472.graph_c0	PREDICTED: uncharacterized protein LOC101883550 [Danio rerio]	4.00	1.46	0.37	0.39	0.30	0.75
c85996.graph_c0	PREDICTED: regulating synaptic membrane exocytosis protein 1 isoform X14 [Danio rerio]	2.22	0.61	0.28	1.62	0.95	0.59
c86852.graph_c0	PREDICTED: uncharacterized protein si:ch211-67e16.4 isoform X3 [Danio rerio]	5.01	1.98	0.39	0.45	3.46	7.69
c86976.graph_c0	PREDICTED: uncharacterized protein LOC101734921 [Xenopus (Silurana) tropicalis]	2.47	0.40	0.16	0.42	0.66	1.56
c88183.graph_c1	PREDICTED: aminoacylase-1 isoform X2 [Danio rerio]	18.90	8.61	0.46	11.05	9.70	0.88
c88340.graph_c0	PREDICTED: uncharacterized protein LOC100332306 isoform X1 [Danio rerio]	8.74	0.05	0.01	1.48	0.05	0.03
c88833.graph_c0	bcl-2-like protein 13 [Danio rerio]	13.08	5.37	0.41	10.43	6.69	0.64
c89899.graph_c0	PREDICTED: uncharacterized protein LOC103911441 [Danio rerio]	2.61	0.81	0.31	2.01	1.39	0.69
c90212.graph_c0	PREDICTED: proactivator polypeptide-like isoform X1 [Danio rerio]	91.17	26.48	0.29	69.29	13.89	0.20
c90547.graph_c0	Pdc1 protein, partial [Danio rerio]	2.24	0.15	0.07	0.60	0.13	0.22
c90965.graph_c0	unnamed protein product [Oncorhynchus mykiss]	1.86	0.42	0.23	0.80	0.45	0.57
c90978.graph_c0	unnamed protein product [Oncorhynchus mykiss]	2.53	0.57	0.23	0.13	0.36	2.77
c91034.graph_c0	PREDICTED: G-protein coupled bile acid receptor 1-like [Danio rerio]	3.82	0.94	0.25	1.28	1.09	0.86
c91501.graph_c1	A-kinase anchor protein 8-like [Danio rerio]	10.87	4.78	0.44	6.35	3.02	0.48

c91953.graph_c0	PREDICTED: uncharacterized protein LOC103475547 [Poecilia reticulata]	3.20	1.02	0.32	2.02	0.50	0.25
c92283.graph_c0	PREDICTED: coiled-coil domain-containing protein 141 [Danio rerio]	3.18	1.40	0.44	1.82	1.28	0.70
c92913.graph_c1	unnamed protein product, partial [Oncorhynchus mykiss]	1.62	0.48	0.30	0.35	0.78	2.25
c93105.graph_c0	PREDICTED: ATPase family, AAA domain containing 5b isoform X1 [Danio rerio]	5.48	2.26	0.41	1.64	2.19	1.34
c94058.graph_c0	potassium voltage-gated channel, Shaw-related subfamily, member 3b [Danio rerio]	4.49	2.05	0.46	3.56	2.44	0.69
c94070.graph_c0	PREDICTED: ecto-NOX disulfide-thiol exchanger 1 [Apaloderma vittatum]	36.28	15.54	0.43	24.30	15.99	0.66
c94181.graph_c0	PREDICTED: wolframin [Danio rerio]	12.92	5.24	0.41	4.49	5.82	1.30
c94383.graph_c0	Caspy2 [Danio rerio]	23.40	8.45	0.36	4.73	10.24	2.16
c94634.graph_c1	pol-like protein [Danio rerio]	4.25	1.30	0.31	2.68	1.39	0.52
c95075.graph_c0	PREDICTED: uncharacterized protein LOC104934881 [Larimichthys crocea]	3.86	1.01	0.26	0.27	1.57	5.77

Table S6 Specific primers used for quantitative PCR validation

Gene description	Gene ID	F/R	qPCR Primer Sequence
gamma-glutamylcyclotransferase	c74062.graph_c0	F	GAGAATGTGAAAATGGGGACT
precursor		R	AGATAATGTGGAGAAGGTGGC
homeobox protein Hox-D3a	c66549.graph_c0	F	ATGTTCAACCTCGGTCATCTTC
		R	GTCTGTGTATGTGGGGTGCG
dnaJ homolog subfamily A member	c80217.graph_c0	F	GCAAGGATTTCCCCCTACCA
1		R	CTGGTTCCTCATCAGACGCTT
deoxynucleoside triphosphate	c68657.graph_c0	F	GTGTTGAAAGAGCACGGACTG
triphosphohydrolase SAMHD1-like		R	AACTGTTACGAATGCCGAGGT
MAC/Perforin domain	c76677.graph_c0	F	GAGGGGTTTCAGAGAGTAGTGC
		R	ATGAGCGGGCTTACAGACACT
Heat shock protein HSP 90-alpha 1	c71586.graph_c0	F	TAAGGGTGTTGTGGACTCTGAGG
		R	GACCAGGTTCTTGCGGATGAC
C-C chemokine receptor type 6	c78819.graph_c0	F	CACGCACTGCCTTGTGTCTCT
		R	CTCCTCTCCCTTCCCACCTTT
chloride intracellular channel	c40508.graph_c0	F	CTGCTGTACGGTACAGAGGTG
protein 1		R	TGTTAGATTCAGGATTGCGAG

Figure.S1 Thermal tolerance polygon of the Hangzhou population (H, marked with dotted line) and Gaizhou population (G, marked with full line) in *Rhynchocypris oxycephalus*.

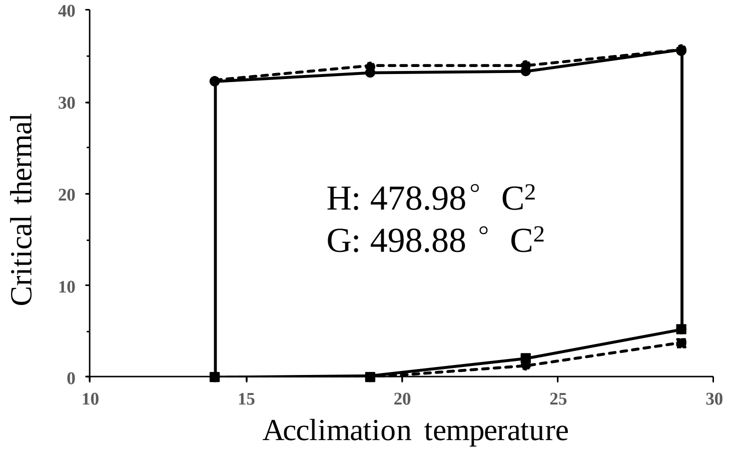

Figure. S2 Quantitative real-time PCR confirmation of differentially expressed genes identified by RNA-seq in a) Hangzhou population; b) Gaizhou population. Log-fold changes are expressed as the ratio of gene expression after normalization to β -actin.

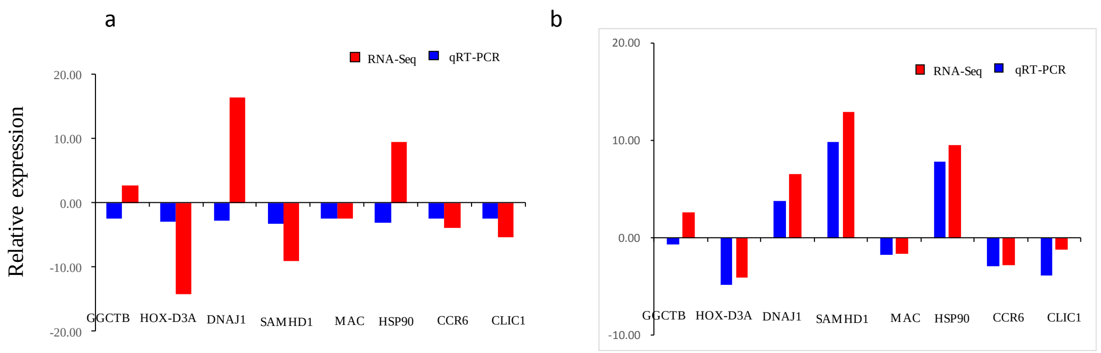

Figure. S3 PCA components 1 and 2 (x and y axis, respectively) of expression values for all 155,636 unigenes in the reference assembly for all samples. The numbers in parentheses represent the proportion of variance explained by that principal component. H represents Hangzhou population and G represents Gaizhou population.

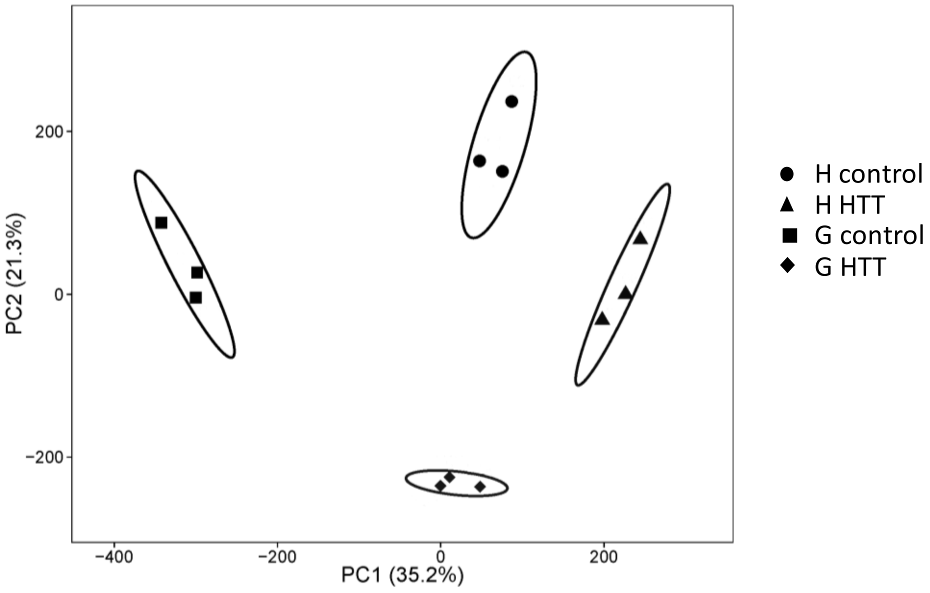

Supplement: Supplementary file 1 — Supplementary Information [file 41598_2018_30074_MOESM1_ESM.pdf]
